# Autocrine Netrin‐1 Signaling in Hepatic Stellate Cells Drives Liver Fibrosis and Diet‐Induced Metabolic Dysfunction‐Associated Steatohepatitis in Mice

**DOI:** 10.1002/advs.202514545

**Published:** 2026-01-09

**Authors:** Jiahui Zhao, Yajie Peng, Hongyan Lei, Tianyi Wang, Huajuan Wang, Bo Wang, Jin Li, Xiaoying Li, Xuelian Xiong

**Affiliations:** ^1^ Ministry of Education Key Laboratory of Metabolism and Molecular Medicine Department of Endocrinology and Metabolism Zhongshan Hospital Fudan University Shanghai China; ^2^ State Key Laboratory of Genetic Engineering School of Life Sciences Fudan University Shanghai China; ^3^ Department of Endocrinology Xinhua Hospital Affiliated to Shanghai Jiao Tong University School of Medicine Shanghai China

**Keywords:** autocrine signaling, hepatic stellate cells, MASH, Netrin‐1

## Abstract

Liver fibrosis is a central feature of progressive liver diseases, including metabolic dysfunction‐associated steatohepatitis (MASH). The profibrotic liver microenvironment drives hepatic stellate cell (HSC) activation and collagen deposition. However, the nature of HSC‐mediated autocrine signaling during the fibrotic response has not been completely characterized. Here, we identify Netrin‐1 as an autocrine factor that drives HSC activation and liver fibrosis in patients with MASH. Hepatic Netrin‐1 expression was consistently elevated across multiple experimental models of liver fibrosis. Functional studies showed that adenovirus‐associated virus (AAV)‐mediated hepatic Netrin‐1 overexpression exacerbated fibrosis, whereas HSC‐specific conditional ablation of Netrin‐1 markedly attenuated diet‐induced MASH and CCl4‐induced liver fibrosis. Notably, lipid nanoparticle‐mediated siRNA knockdown of Netrin‐1 ameliorated liver fibrosis in mice. Mechanistic investigations revealed that Netrin‐1 promotes HSC activation through autocrine signaling mediated by the UNC5B receptor, which triggers rapid intracellular Ca^2+^ mobilization and downstream SMAD2 phosphorylation and fibrogenic gene expression. Collectively, our findings identify a novel autocrine signaling axis in which HSC‐derived Netrin‐1 establishes a positive feedback loop that sustains HSC activation and drives fibrotic progression. Blocking the Netrin‐1‐mediated fibrogenic response may offer a potential therapeutic strategy for anti‐fibrotic interventions.

## Graphical abstract

Netrin‐1 expression is upregulated in hepatic stellate cells (HSCs) during metabolic dysfunction–associated steatohepatitis and injury‐mediated liver fibrosis. Secreted Netrin‐1 establishes an autocrine positive feedback loop by binding to UNC5B receptors on HSCs. Receptor activation triggers calcium influx and profibrotic response in HSCs, leading to increased extracellular matrix (ECM) production and liver fibrosis.

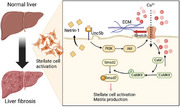

**Abbreviations**
6‐OHDA: 6‐hydroxydopamine
AAV: adenovirus‐associated virus
ALT: alanine aminotransferase
AST: aspartate aminotransferase
BDL: bile duct ligation
CaMK II: calcium/calmodulin‐dependent protein kinase II
CCl4: carbon tetrachloride
DCC: deleted in colorectal cancer
DDC: 3,5‐diethoxycarbonyl‐1,4‐dihydrocollidine
DMEM: dulbecco's modified eagle medium
ECM: extracellular matrix
ES: embryonic stem
FBS: fetal bovine serum
H&E: hematoxylin & eosin
HCC: hepatocellular carcinoma
HFD: high‐fat diet
HFMCD: high‐fat, methionine‐ and choline‐deficient diet
HSCs: hepatic stellate cells
KO: knockout
LNP: lipid nanoparticle
MASH: metabolic dysfunction‐associated steatohepatitis
MASLD: metabolic dysfunction‐associated steatotic liver disease
NPCs: nonparenchymal cells
NTN: Netrin protein
PBS: phosphate‐buffered saline
qPCR: quantitative polymerase chain reaction
SV40 T: simian virus 40 large T antigen
TBG: thyroxine binding globulin
TGF: transforming growth factor
UMAP: uniform manifold approximation and projection
α‐SMA: alpha‐smooth muscle actin

## 1 Introduction

Metabolic dysfunction‐associated steatotic liver disease (MASLD) has emerged as a significant global public health challenge, driven by its rising prevalence [1, 2]. MASLD encompasses various liver pathologies, ranging from simple steatosis to more severe forms such as metabolic dysfunction‐associated steatohepatitis (MASH), which is characterized by significant liver injury and fibrosis [3]. Liver fibrosis is a central histological feature of advanced MASLD. The degree of fibrosis strongly correlates with morbidity and mortality in these patients and is a key predictor of severe liver‐related outcomes, including cirrhosis and hepatocellular carcinoma (HCC) [4, 5]. Current therapeutic strategies for reversing liver fibrosis are limited, highlighting the urgent need for effective antifibrotic treatments targeting the underlying pathogenic mechanisms of fibrogenesis.

The initiation and progression of liver fibrosis involve multiple signaling pathways and cellular interactions [6, 7, 8]. It is marked by excessive accumulation of extracellular matrix (ECM) components, primarily mediated by hepatic stellate cells (HSCs). Under normal physiological conditions, HSCs are quiescent and store vitamin A; however, chronic liver injury activates them into myofibroblast‐like cells that produce collagen and other fibrogenic factors [6]. Recent single‐cell transcriptomic analyses have highlighted the spatial heterogeneity and cell‐state transitions of HSCs in fibrotic liver disease [9, 10, 11]. HSCs can be classified into portal and central‐vein‐associated subtypes based on their transcriptional and topological features [9]. During liver fibrosis, these cells transition from a quiescent to an activated phenotype, marked by increased expression of genes involved in ECM deposition and remodeling [10, 11]. Interestingly, activated HSCs retain the capacity to revert to a quiescent‐like state following resolution of the underlying liver disease, indicating that liver fibrosis is reversible under certain conditions. In liver biology, HSCs play a pivotal role in producing ECM components and mediating communication between different liver cell types through paracrine and autocrine signaling mechanisms [12]. Activated HSCs secrete a variety of cytokines that influence neighboring hepatocytes, immune cells, and endothelial cells [6, 13, 14, 15]. Recent studies have shown that autocrine signaling circuits within HSCs contribute to their activation and fibrosis progression [16].

Netrin proteins (NTN) are an evolutionarily conserved family of genes that comprises a group of extracellular proteins that play key roles in guiding cell and axon migration during embryonic development [17]. In mammals, three netrins (Netrin‐1, Netrin‐3, and Netrin‐4) and two glycosylphosphatidylinositol‐anchored membrane proteins (Netrin‐G1 and G2) have been identified [18]. These proteins function as bifunctional signals, attracting some cell types while repelling others, thus influencing cell migration and adhesion in different contexts beyond the nervous system, including organogenesis in the lungs, pancreas, and vasculature [19, 20, 21, 22, 23, 24]. Moreover, Netrin signaling has been implicated in the regulation of angiogenesis, tumorigenesis, and the tissue microenvironment [25, 26, 27, 28, 29, 30]. However, the pathophysiological role of netrins in fibrotic liver disease and whether this pathway is amenable to therapeutic intervention remains inadequately explored. In this study, we identified Netrin‐1 as an HSC‐derived secreted factor activated by diet‐induced MASH. We demonstrated that Netrin‐1‐mediated autocrine signaling substantially contributes to liver fibrosis during MASH pathogenesis, and that nanoparticle‐mediated inhibition of Netrin‐1 ameliorates fibrosis.

## 2 Results

### 2.1 Identification of Netrin‐1 as an HSC‐Enriched Secreted Factor

**FIGURE 1:**
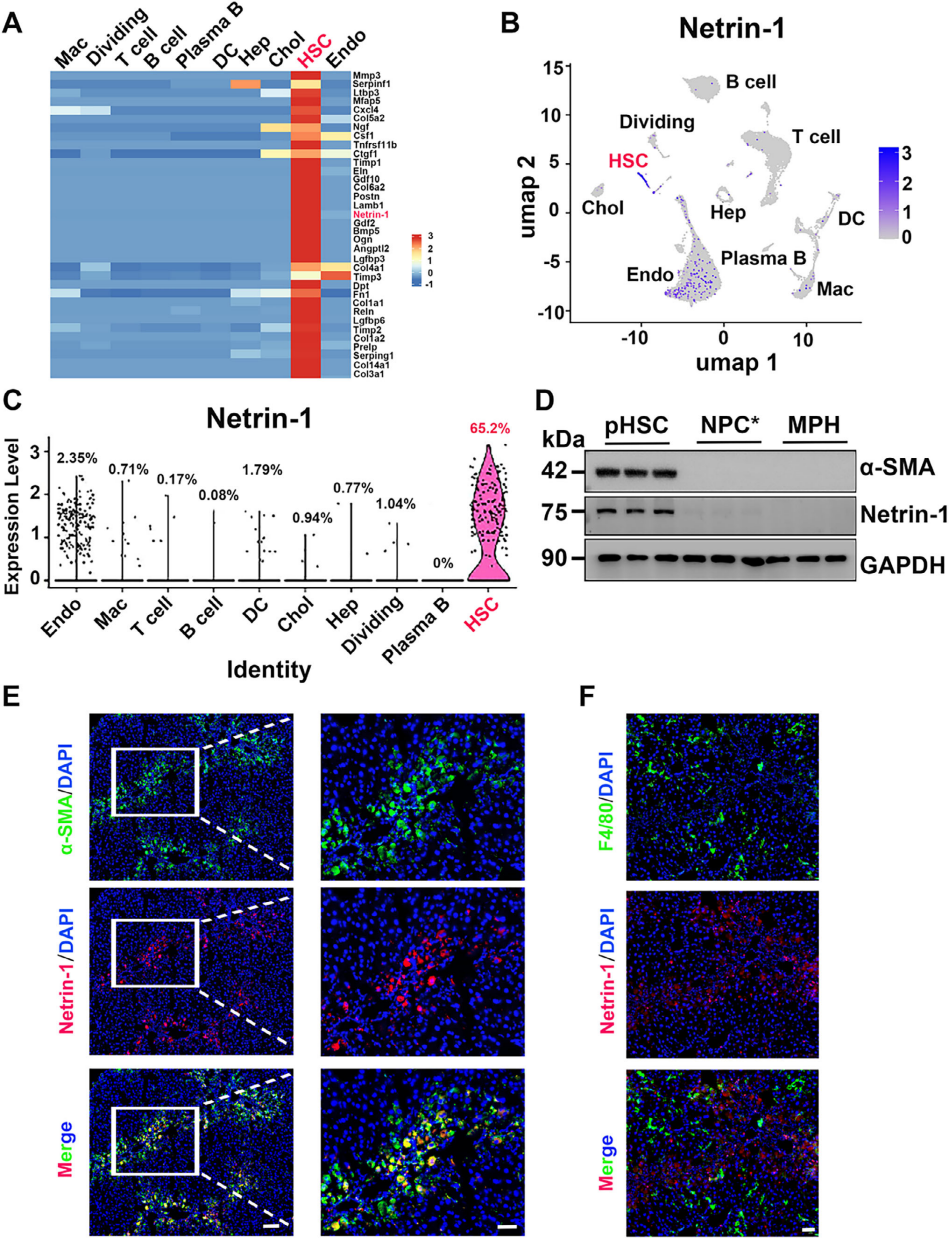
Netrin‐1 is a HSC‐enriched secreted factor in the liver. (A) Heatmap of HSC‐enriched genes encoding secreted factors in the liver (GSE129516). (B) UMAP plots showing relative Ntn‐1 mRNA expression across liver cell types. (C) Violin plots of Ntn‐1 gene expression. (D) Immunoblotting of isolated mouse primary hepatocytes (MPH), primary HSCs (pHSCs), and NPCs with pHSCs depleted (NPC^*^). (E,F) Immunofluorescence staining for Netrin‐1, α‐SMA, and F4/80 in mouse liver. Representative images. Scale bar: 100 µm.

Intercellular communication among various liver cell types is essential for maintaining tissue homeostasis and metabolic function. Disruptions in paracrine and autocrine ligand‐receptor signaling contribute to key aspects of metabolic liver disease progression, including hepatocyte injury, inflammatory responses, and liver fibrosis [31, 32]. Recent single‐cell secretome gene expression and ligand‐receptor pairing analysis have outlined a framework for cell–cell communication in mouse and human livers [12]. HSCs are best known for secreting ECM proteins during liver fibrosis; nevertheless, the full spectrum of HSC‐derived secreted factors and their roles in intrahepatic signaling remain unclear. Therefore, we analyzed our single‐cell RNA‐seq dataset on non‐parenchymal cells (NPCs) and identified an HSC‐enriched gene set encoding ECM structural proteins (e.g., Col1a1, Col3a1, and Fn1), matrix remodeling proteins (e.g., Mmp3, Timp1, and Timp2), and growth factors (e.g., Ngf, Gdf2, and Bmp5) (Figure 1A). Interestingly, Netrin‐1 showed prominent mRNA expression within the HSC cluster, as demonstrated using Uniform Manifold Approximation and Projection (UMAP) and violin plots (Figure 1B–C). Netrin‐1 mRNA expression was low across most liver cell types and was detected only in a subset of endothelial cells (2.35%); however, over 65% of HSCs exhibited high Netrin‐1 mRNA expression in the liver.

To confirm cell type‐specific Netrin‐1 expression, we fractionated dissociated liver cells into hepatocytes, HSCs, and HSC‐depleted NPCs (NPC^*^). Immunoblotting analysis revealed that Netrin‐1 protein was detected almost exclusively in the HSCs fraction, but not in hepatocytes or HSC‐depleted NPCs (Figure 1D). Immunofluorescence studies on the liver sections from carbon tetrachloride (CCl4)‐treated mice further showed that Netrin‐1 expression was colocalized with alpha‐smooth muscle actin (α‐SMA), a marker of activated HSCs (Figure 1E). Conversely, we observed no appreciable co‐localization of Netrin‐1 and F4/80, a marker for liver macrophages (Figure 1F). Collectively, these findings indicate that HSCs are the primary source of Netrin‐1 in the fibrotic liver microenvironment.

### 2.2 Netrin‐1 Expression is Elevated in Metabolic Liver Disease and Liver Fibrosis

**FIGURE 2:**
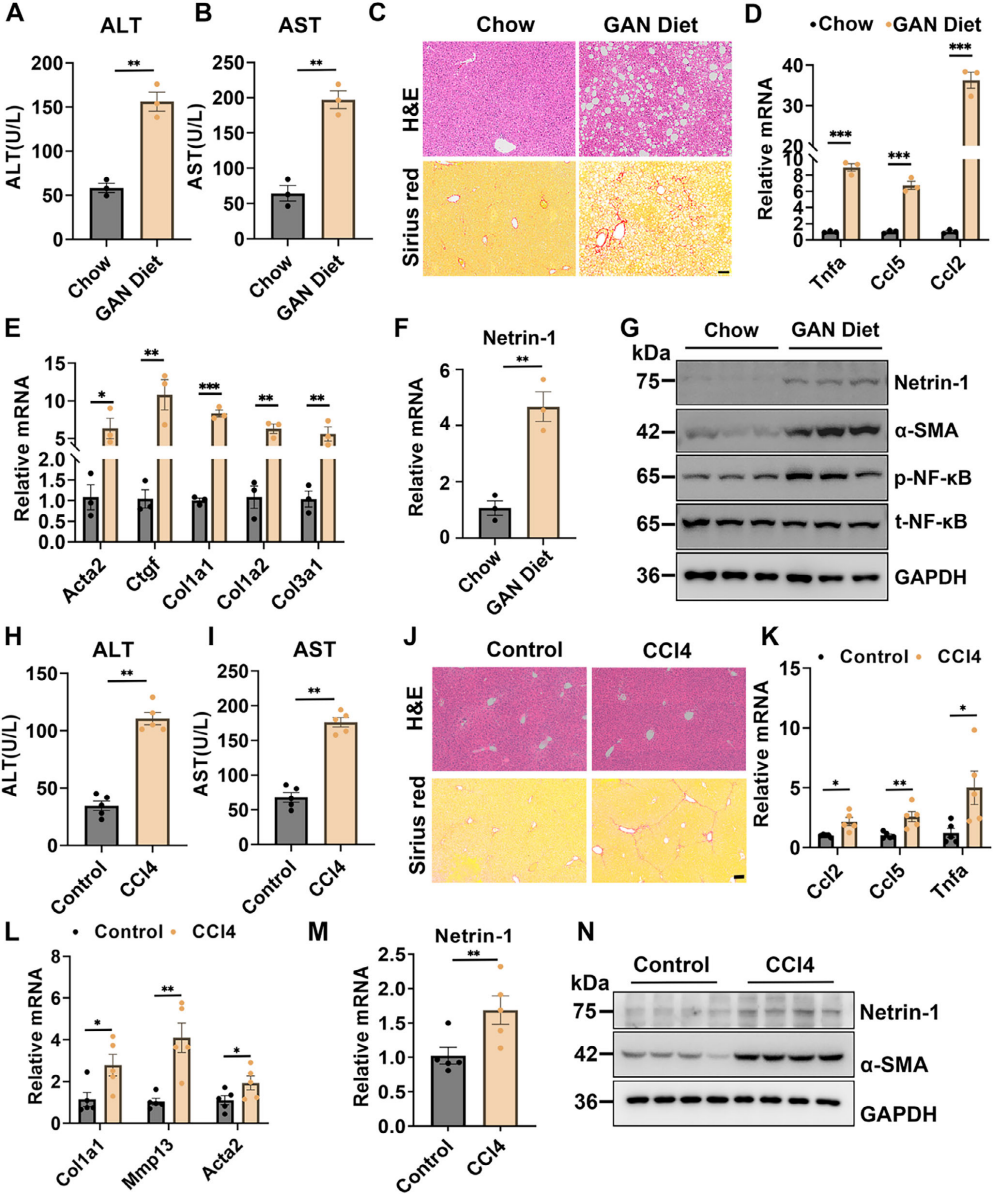
Induction of Netrin‐1 expression in MASH and fibrotic liver. Mice were fed a chow (n = 3) or GAN (n = 3) diet for 5 months. (A, B) Plasma ALT and AST levels. (C) H&E (top) and Sirius Red (bottom) staining of liver sections (scale bar = 100 µm). (D–F) qPCR analysis of hepatic gene expression. (G) Immunoblotting of total liver lysate. (H‐N) Mice received i.p. injections of vehicle or CCl4 (0.6 ml/kg body weight) twice weekly for 3 weeks and were sacrificed 4 days after the final injection (n = 5). (H, I) Plasma ALT and AST levels. (J) H&E (top) and Sirius Red (bottom) staining of liver sections (scale bar = 100 µm). (K, L) qPCR analysis of hepatic gene expression. (M) qPCR analysis of Nnt1 expression. (N) Immunoblotting of total liver lysate. Data are shown as means ± SEM. * p < 0.05, ** p < 0.01, *** p < 0.001; two‐tailed unpaired Student's *t*‐test.

Netrin‐1 belongs to a family of secreted laminin‐related proteins that play crucial roles in axonal guidance, cell migration, and tissue morphogenesis. Netrin‐1 signaling has recently been implicated in lung fibrosis [19]. However, its role in HSC biology in metabolic liver disease remains unexplored. The prominent expression of Netrin‐1 in HSCs suggests that it may contribute to the development of liver fibrosis. To explore this, we first examined Netrin‐1 expression in different diet‐induced MASH and CCl4‐induced liver fibrosis models. Mice developed characteristic MASH pathology following 5 months on the GAN diet. Compared with chow control, GAN‐fed mice showed significantly elevated plasma alanine transaminase (ALT) and aspartate aminotransferase (AST) levels (Figure 2A,B) and developed marked hepatic steatosis that was associated with inflammatory cell infiltration and liver fibrosis, as demonstrated using hematoxylin and eosin (H&E) and Sirius Red staining (Figure 2C). Quantitative polymerase chain reaction (qPCR) analysis of hepatic gene expression revealed that Netrin‐1 mRNA levels significantly increased upon diet‐induced MASH, along with those of genes associated with inflammation (Tnfa, Ccl5, and Ccl2) and liver fibrosis (Acta2, Ctgf, Col1a1, Col1a2, and Col3a1) (Figure 2D–F). Consistently, Netrin‐1and α‐SMA (encoded by Acta2) protein levels were markedly elevated in MASH livers (Figure 2G). The livers of mice fed a high‐fat methionine‐choline‐deficient diet (HFMCD) for 8 weeks (Figure S1A–G), and mice fed a high‐fat diet (HFD) for 8 months (Figure S1H–M) exhibited similar induction of Netrin‐1 mRNA and protein expression. Netrin‐1 expression was not significantly elevated in livers with steatosis but no fibrosis after 2 months of HFD feeding, suggesting that increased Netrin‐1 levels are specifically associated with progressive fibrotic stages. Subsequently, we examined hepatic Netrin‐1 expression during CCl4‐induced liver injury and fibrosis. As expected, CCl4‐treated mice developed significant liver injury, inflammation, and fibrosis (Figure 2H–L), accompanied by significant elevation of Netrin‐1 mRNA and protein levels (Figure 2M,N). We also observed elevated hepatic Netrin‐1 expression in other liver fibrosis models beyond MASH, including bile duct ligation (BDL)‐induced fibrosis model (Figure S2A–D) and 3,5‐diethoxycarbonyl‐1,4‐dihydrocollidine (DDC) diet‐induced fibrosis models (Figure S2E–J). These results showed that an elevated hepatic Netrin‐1 level is a common feature of fibrotic liver diseases.

### 2.3 Netrin‐1 Exacerbates Diet‐Induced MASH Progression

**FIGURE 3:**
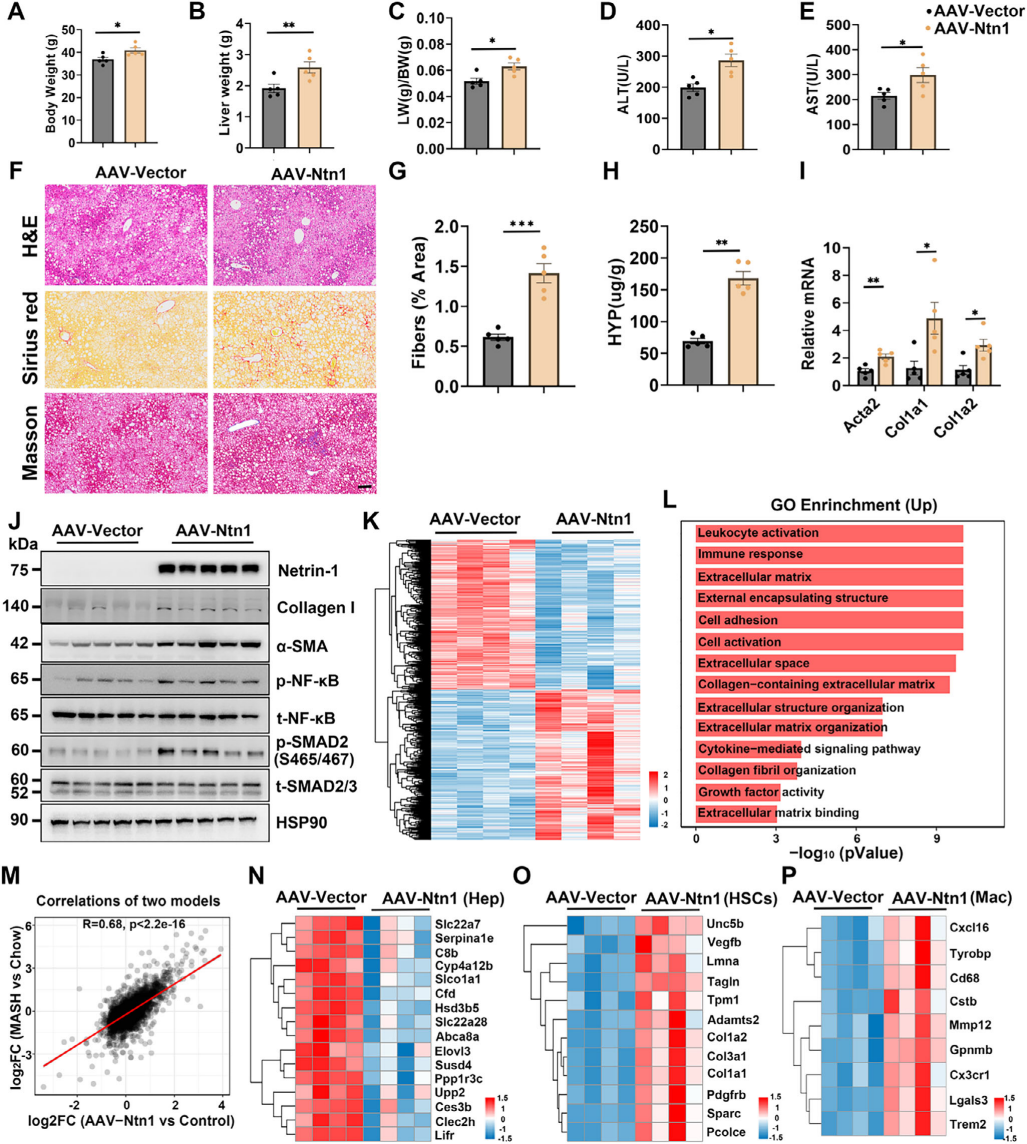
AAV‐mediated overexpression of Netrin‐1 exacerbates liver fibrosis. AAV8‐TBG‐Vector (AAV‐Vector) and AAV8–TBG‐Ntn1(AAV‐Ntn1) were administered prior to GAN diet feeding. The following parameters were measured in AAV‐vector (n = 5) and AAV‐Ntn1 mice (n = 5) fed a GAN diet for 4 months: (A–C) Body weight, liver weight, and liver‐to‐body weight ratio in transduced mice. (D, E) Plasma ALT and AST levels. (F) H&E (top), Sirius Red (middle), and Masson's trichrome staining (bottom) of liver sections (scale bar = 100 µm). (G) Quantification of Sirius Red‐positive areas in liver sections. (H) Liver hydroxyproline content. (I) qPCR analysis of hepatic gene expression. (J) Immunoblotting of total liver lysate. (K) Heatmap of differentially expressed genes in the liver. (L) Gene ontology analysis of upregulated genes in AAV‐Ntn1 mice. (M) Correlation of differentially expressed genes in diet‐induced MASH livers (GSE129516) versus Ntn1 overexpression. (N–P) Heatmaps of hepatocyte‐, HSC‐, and macrophage‐enriched genes regulated by Ntn1 overexpression. Data are presented as mean ± SEM. *p < 0.05, **p < 0.01; two‐tailed unpaired Student's *t*‐test.

Disease‐associated Netrin‐1 induction during diet‐induced MASH and CCl4‐induced liver fibrosis suggests a potential pathogenic role in liver disease progression. To investigate this, we generated a recombinant AAV vector under the control of the thyroxine‐binding globulin (TBG) promoter, which directs liver‐specific Netrin‐1 overexpression. Hepatocytes efficiently expressed the transgene and secreted Netrin‐1 into the extracellular space, increasing its concentration in the hepatic microenvironment surrounding HSCs. To assess hepatocyte‐derived Netrin‐1 secretion, we isolated primary hepatocytes from mice injected with AAV‐TBG‐Vector or AAV‐TBG‐Ntn1 and cultured them in vitro. Analysis of the culture medium revealed substantially elevated Netrin‐1 levels, indicating efficient secretion by hepatocytes upon overexpression (Figure S3A, B). Conditioned medium from Netrin‐1‐overexpressing hepatocytes further enhanced mHSCs activation in vitro, as shown by increased expression of fibrogenic markers compared with control hepatocyte medium (Figure S3C). Mice were transduced with either control AAV‐TBG‐Vector or AAV‐TBG‐Ntn1 and fed a GAN diet for 4 months. While body weight was similar between the two groups, liver weight and plasma liver injury markers were slightly higher in mice with hepatic Netrin‐1 overexpression (Figure 3A–E). Histological analysis showed that AAV‐mediated Netrin‐1 overexpression exacerbated liver fibrosis development, as indicated using Sirius red and Masson's trichrome staining (Figure 3F,G) and quantitative measurements of liver hydroxyproline content (Figure 3H). Conversely, Netrin‐1 did not influence hepatic steatosis in transduced mice. Hepatic gene expression analysis revealed that Netrin‐1 overexpression increased mRNA levels of fibrosis markers, including Acta2, Col1a1, and Col1a2 (Figure 3I). Protein analysis further confirmed elevated levels of Collagen I and α‐SMA in response to AAV‐mediated Netrin‐1 overexpression. Phosphorylation of SMAD2, a key mediator of transforming growth factor (TGF)‐β signaling, was stimulated by Netrin‐1, while total SMAD2 protein levels were comparable between the two groups (Figure 3J). Although hepatocyte‐specific overexpression of Netrin‐1 is insufficient to initiate HSC activation or fibrosis (Figure S4), its profibrotic role was also observed in both HFMCD‐ and long‐term HFD‐induced MASH models (Figure S5A–H).

To investigate how Netrin‐1 influences global gene expression in the liver, we performed RNA‐seq analysis on mice transduced with AAV‐ Vector or AAV‐Ntn1 (Figure 3K). Upregulated genes were enriched in pathways related to immune response, ECM biology, and cytokine signaling (Figure 3L). Correlation analysis revealed that hepatic Netrin‐1 overexpression promoted global gene expression patterns closely resembling those observed in diet‐induced MASH livers (Figure 3M). We next examined the effect of Netrin‐1 on the expression of genes enriched in individual liver cell types. Based on our previous single‐cell RNA‐seq analysis of healthy and MASH livers, we identified gene sets with enriched expression in individual liver cell types [12]. As shown in Figure 3N–P, many hepatocyte‐enriched genes were downregulated by Netrin‐1, whereas genes enriched in HSCs and macrophages exhibited increased mRNA levels. Particularly, several molecular markers of Trem2+ macrophages, including Trem2, Gpnmb, and Mmp12, were elevated in livers from AAV‐Ntn1‐transduced mice. These results indicate that Netrin‐1 promotes a pro‐fibrotic response in the liver and exacerbates diet‐induced MASH progression.

Subsequently, we investigated whether Netrin‐1 promotes the progression of liver fibrosis in CCl4‐treated mice. Mice transduced with AAV‐TBG‐Vector or AAV‐TBG‐Ntn1 were treated with CCl4 twice weekly for 2 weeks. Histological staining showed more pronounced bridging fibrosis in the livers of AAV‐TBG‐Ntn1‐transduced mice than in the livers of control mice (Figure S5I). Hepatic hydroxyproline content and expression of fibrosis‐associated genes were significantly increased by Netrin‐1 (Figure S5J,K), and protein analysis of profibrotic signaling and ECM proteins further confirmed these findings (Figure S5L). Collectively, these results support a profibrotic role for Netrin‐1 in the context of diet‐induced MASH and CCl4‐induced liver fibrosis.

### 2.4 HSC‐Specific Inactivation of Netrin‐1 Mitigates Liver Fibrosis

**FIGURE 4:**
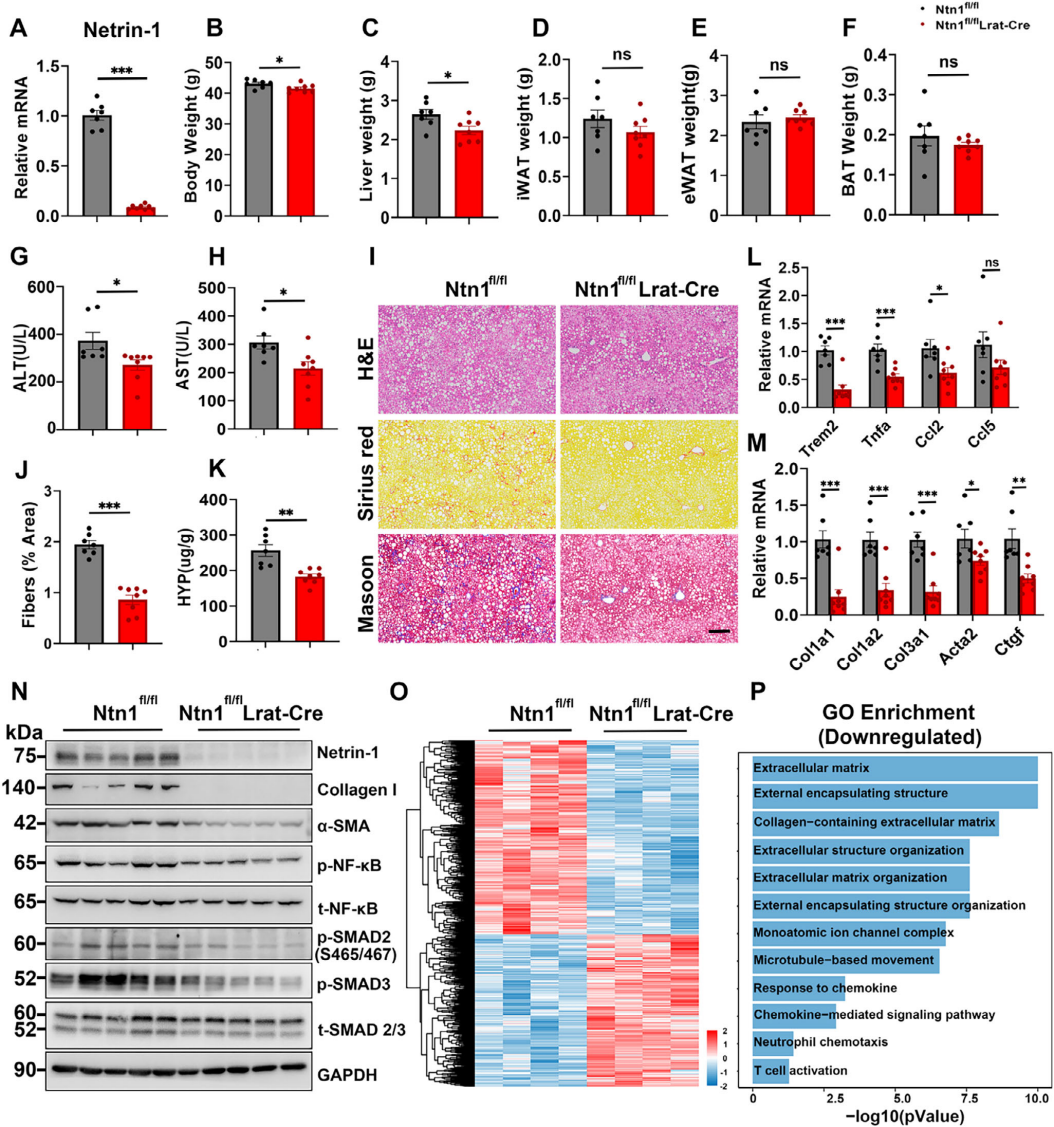
HSC‐specific Netrin‐1 deletion alleviates liver fibrosis following GAN diet feeding.Ntn1^fl/fl^ (n = 7) and Ntn1^fl/fl^ Lrat‐Cre (n = 8) mice were fed a GAN diet for 5 months. (A) qPCR analysis of hepatic Ntn1 expression. (B–F) Body and tissue weight. (G, H) Plasma ALT and AST levels. (I) H&E (top), Sirius Red (middle), and Masson trichrome staining (bottom) of liver sections (scale bar = 100 µm). (J) Quantification of Sirius Red‐positive areas in liver sections. (K) Liver hydroxyproline content. (L, M) qPCR analysis of hepatic gene expression. (N) Immunoblotting of total liver lysate. (O) Heat map of differentially expressed genes. (P) Gene ontology analysis of downregulated gene clusters. Data are presented as mean ± SEM. *p < 0.05, **p < 0.01, ***p < 0.001; two‐tailed unpaired Student's *t*‐test.

Whole‐body Netrin‐1 deficiency causes severe neural abnormalities, resulting in embryonic and perinatal lethality in mice [33]. To critically assess the role of Netrin‐1 in mediating HSC activation and liver fibrosis, we generated mice with HSC‐specific Netrin‐1 ablation using Lrat‐Cre, an established genetic tool for conditional gene inactivation in HSCs [34]. As expected, hepatic Netrin‐1 mRNA expression was markedly reduced in Ntn1^fl/fl^Lrat‐Cre mice (Figure 4A). Ntn1^fl/fl^ and Ntn1^fl/fl^Lrat‐Cre mice were then fed a GAN diet for 5 months to determine the contribution of HSC‐derived Netrin‐1 to MASH development. We observed no significant differences in adipose tissue, while plasma ALT & AST levels were significantly lower in HSC‐specific Ntn1 knockout (KO) mice than in controls (Figure 4B–H). HSC‐specific Ntn1 KO mice exhibited markedly reduced liver fibrosis, as assessed using Sirius Red and Masson's trichrome staining, and hydroxyproline quantification (Figure 4I–K). Consistently, qPCR analysis revealed significantly lower expression of genes associated with inflammation (Trem2, Tnfa, Ccl2, and Ccl5) and liver fibrosis (Col1a1, Col1a2, Col3a1, Acta2, and Ctgf) in Netrin‐1‐deficient mouse livers (Figure 4L–M). Immunoblotting analysis demonstrated that HSC‐specific Netrin‐1 inactivation attenuated SMAD2 phosphorylation and reduced expression of protein markers characteristic of liver fibrosis, including Collagen I and α‐SMA (Figure 4N). Transcriptional profiling of control and Ntn1‐deficient mouse livers revealed that HSC‐specific Netrin‐1 inactivation profoundly impacts global hepatic gene expression. Many downregulated genes in Netrin‐1‐deficient livers were associated with ECM biology, fibrotic responses, and immune signaling (Figure 4O–P). These findings indicate that Netrin‐1 plays a critical role in promoting inflammation and fibrogenesis within the MASH liver microenvironment.

We next assessed whether Netrin‐1 is required for advanced liver fibrosis following HFMCD diet and CCl4‐induced liver injury and fibrosis. Body weight, liver weight, and plasma ALT and AST levels were comparable between groups, indicating that Netrin‐1 ablation did not directly affect liver metabolism or HFMCD‐induced hepatocyte injury (Figure S6A–E). Conversely, conditional Ntn1 deletion in HSCs markedly reduced collagen deposition and liver fibrosis after HFMCD‐induced MASH, as shown using histological analysis such as Sirius red and Masson's trichrome staining. (Figure S6F). Consistently, hepatic hydroxyproline content was significantly lower in Ntn1^fl/fl^Lrat‐Cre mice than that in the Ntn1^fl/fl^ group (Figure S6G). Gene expression analysis showed that mRNA expression of key inflammatory and fibrotic markers, including Ccl2, Ccl5, Tnfa, Col1a1, Col1a2, and Acta2, was significantly reduced upon Netrin‐1 ablation (Figure S6H,I). Immunoblotting further revealed decreased protein expression of Collagen I and α‐SMA, along with reduced SMAD2 phosphorylation in the KO group (Figure S6J). In CCl4‐induced liver injury and fibrosis models, we also found that genetic ablation of HSC‐derived Netrin‐1 significantly attenuated liver fibrosis (Figure S6K‐N). These findings underscore the critical role of HSC‐derived Netrin‐1 in the progression of advanced liver fibrosis.

### 2.5 Netrin‐1 Exacerbates Liver Fibrosis Independently of Sympathetic Innervation

Since Netrin‐1 is a well‐established pro‐neuronal migration factor, and hepatic sympathetic nerves play a crucial role in regulating liver fibrosis [35, 36, 37], we next evaluated whether Netrin‐1 promotes liver fibrosis by modulating hepatic sympathetic innervation. To test this, we chemically ablated liver sympathetic nerves using 6‐hydroxydopamine (6‐OHDA) [36, 37] and then subjected the mice to CCl4‐induced liver fibrosis (Figure S7A,B). Short‐term 6‐OHDA treatment did not significantly alter body weight or liver weight but effectively reduced liver enzymes and liver fibrosis, consistent with the known profibrotic role of sympathetic nerve activity (Figure S7C–G). Sirius Red and Masson staining, along with gene expression analysis, showed that AAV‐mediated Netrin‐1 overexpression exacerbated fibrosis in 6‐OHDA‐treated mouse livers, indicating that the profibrotic actions of Netrin‐1 were independent of hepatic sympathetic innervation (Figure S7H–J). Western blot analysis further confirmed that Netrin‐1 activated fibrogenic signaling pathways and increased the expression of ECM proteins, including α‐SMA and Collagen I (Figure S7K). Together, these findings demonstrate that Netrin‐1 drives liver fibrosis via a mechanism distinct from sympathetic nerve regulation.

### 2.6 Netrin‐1 Cell‐Autonomously Promotes Fibrotic Response in HSCs

**FIGURE 5:**
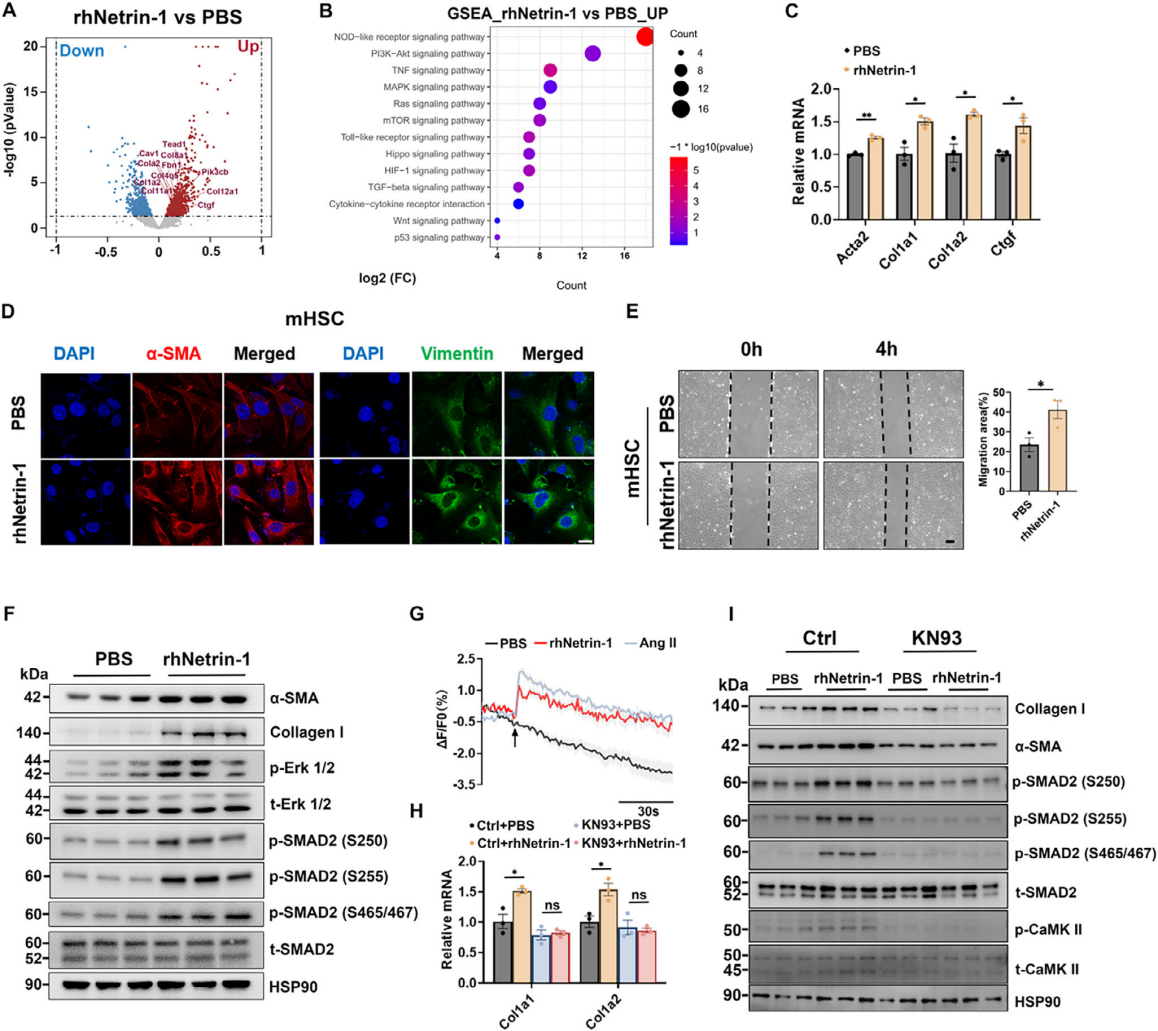
Netrin‐1 promotes fibrotic gene expression by stimulating calcium signaling in HSCs. (A) Volcano plot of differentially expressed genes in mHSCs treated with rhNetrin‐1 protein or PBS for 12 hrs. (B) Gene set enrichment analysis of upregulated genes. (C) qPCR analysis of fibrotic gene expression. (D) Immunofluorescence staining of α‐SMA (red) and Vimentin (green) in mHSCs treated with PBS or rhNetrin‐1 (300 ng/mL) for 24 hrs. Nuclei, DAPI (blue). (E) Wound‐healing assay of mHSCs after rhNetrin‐1 treatment; migration rate quantified on the right. (F) Immunoblotting of total cell lysates 24 hrs post‐treatment. (G) Calcium imaging traces in mHSCs treated with PBS (n = 6), Netrin‐1 (300ng/ml, n = 6), angiotensin II (10ng/ml, n = 9). Arrows indicate treatment initiation. Data are presented as mean ± SD. (H) Gene expression analysis of mHSCs pretreated with or without 10µM KN93, followed by PBS or Netrin‐1 treatment. (I) Immunoblotting of total cell lysates. Data are presented as mean ± SEM. *p < 0.05, **p < 0.01; two‐tailed unpaired Student's *t*‐test.

As described above, Netrin‐1 expression was largely restricted to HSCs in the liver. Based on the observation that AAV‐mediated Netrin‐1 overexpression exacerbates liver fibrosis with only modest inflammatory changes and no significant effect on steatosis, we proposed the possibility of direct autocrine action of Netrin‐1 in HSCs. We next performed Netrin‐1 treatments in cultured mHSCs, followed by bulk RNA‐seq analysis to delineate its cell‐autonomous effects and underlying signaling mechanisms. Differential gene expression analysis revealed 425 upregulated and 259 downregulated genes after Netrin‐1 treatment (Figure 5A). Pathway enrichment analysis showed that the upregulated genes, including Col1a2, Ctgf, Col1a1, Fbn1, and Tead1, were associated with fibrosis and inflammation (Figure 5B). The upregulation of profibrotic gene expression was further validated by qPCR (Figure 5C). Immunofluorescence staining showed that Netrin‐1 treatment markedly increased α‐SMA and Vimentin expression in mHSCs (Figure 5D). Furthermore, wound healing assays demonstrated that Netrin‐1‐treated mHSCs exhibited significantly enhanced migratory capacity compared with controls (Figure 5E), further supporting its role in promoting the activated phenotype. Notably, Netrin‐1 treatment stimulated ERK and SMAD2 phosphorylation in cultured mHSCs, as well as ECM protein production (Figure 5F). Previous studies have shown that activated HSCs elicit a robust increase in intracellular Ca^2+^ levels in response to vasoactive hormones, such as angiotensin II (Ang II) [12, 38, 39]. Consistently, we observed a rapid rise in intracellular Ca^2+^ levels in mHSCs following Netrin‐1 exposure (Figure 5G). This rapid calcium mobilization suggests that Netrin‐1 may modulate HSC activity and function through calcium‐dependent signaling pathways. Because calcium/calmodulin‐dependent protein kinase II (CaMKII) is a key mediator of calcium signaling across diverse cell types [39, 40, 41], we next tested whether CaMKII activation is required for the profibrotic actions of Netrin‐1 by treating cultured mHSCs with phosphate‐buffered saline (PBS) or Netrin‐1 in the absence or presence of KN93, a potent CaMKII inhibitor. KN93 nearly abolished Netrin‐1‐induced SMAD2 phosphorylation and the expression of fibrosis‐associated genes (Figure 5H,I). Furthermore, we corroborated these observations in a human hepatic stellate cell line LX2 (Figure S8).

### 2.7 UNC5B Mediates Profibrotic Signaling by Netrin‐1 in HSCs

**FIGURE 6:**
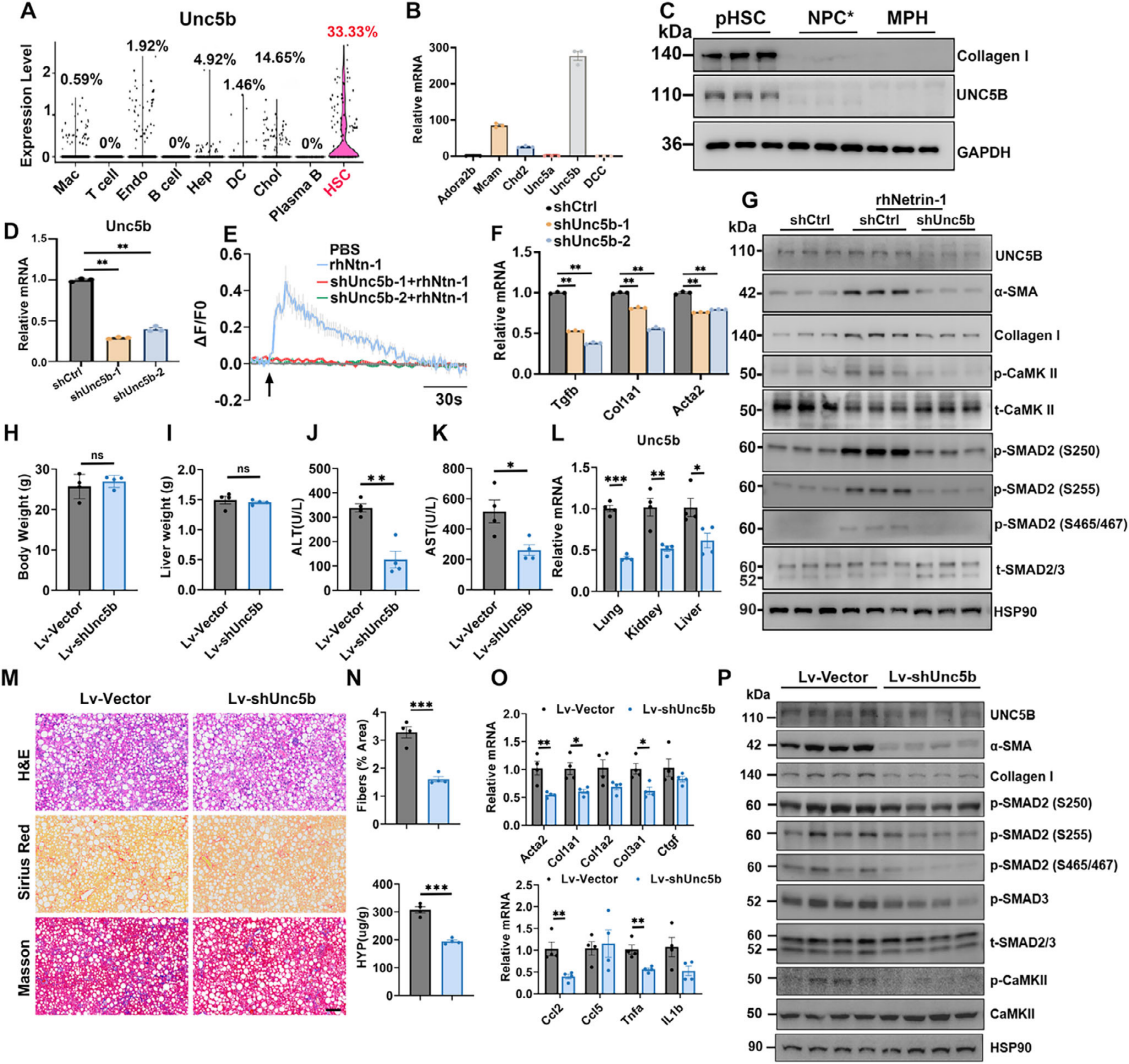
UBC5B is required for HSC activation in response to Netrin‐1. (A) Violin plots showing Unc5b gene expression in each liver cell cluster (GSE129516). (B) qPCR analysis of Netrin‐1 receptor gene expression in mHSCs. (C) Immunoblotting of isolated pHSC, MPH, and NPC*. (D–F) mHSCs were transduced with lentivirus‐carrying vectors containing the indicated shRNAs or an empty vector (shCtrl). (D) qPCR analysis of Unc5b expression. (E) Calcium imaging traces in mHSCs treated with phosphate buffer saline or Netrin‐1. (F) qPCR analysis of fibrosis‐related gene expression. (G) Immunoblotting of total cell lysates. (H–P) Eight‐week‐old C57BL/6 mice were injected with 1 x 10^8 TU/mouse lentivirus particles via the tail vein, followed by 2‐month HFMCD feeding (n = 4 per group). (H,I) Body and liver weight. (J, K) Serum ALT and AST levels. (L) Relative Unc5b expression in lung, kidney, and liver tissues. (M) H&E (top), Sirius Red (middle), and Masson trichrome staining (bottom) of liver sections (scale bar = 100 µm). (N) Quantification of Sirius Red‐positive areas in liver sections and hydroxyproline content in liver tissue. (O) qPCR analysis of hepatic fibrosis‐ and inflammation‐related genes in liver tissues. (P) Immunoblotting of total liver lysate. Data are presented as mean ± SEM. *p < 0.05, **p < 0.01; two‐tailed unpaired Student's *t*‐test.

The Netrin family of ligands exerts diverse biological effects on axon guidance, branching, and cell migration by engaging different cellular receptors, particularly UNC5 receptors and Deleted in Colorectal Cancer (DCC) [20, 42, 43]. Analysis of our previous liver single‐cell RNA‐seq dataset showed that Unc5b was most prominently expressed in HSCs (Figure 6A), whereas the mRNA levels of Dcc, Unc5a, and Unc5c were comparatively low in the liver (Figure 6B). Immunoblotting analysis confirmed that UNC5B protein expression was detected exclusively in HSCs and absent in HSC‐depleted non‐parenchymal cell types and hepatocytes (Figure 6C). Collagen I was used as an HSC marker protein and served as a positive control for liver cell fractionation. To evaluate the functional role of UNC5B in HSCs, we performed shRNA knockdown using two independent target sequences and found that silencing UNC5B markedly reduced the Netrin‐1‐induced calcium response in mHSCs (Figure 6D, E). UNC5B knockdown also diminished Netrin‐1‐induced CaMKII and SMAD2 phosphorylation, resulting in decreased expression of genes associated with HSC activation and fibrosis, including Tgfb, Col1a1, and Acta2 (Figure 6F, G). These findings indicate that UNC5B is essential for mediating Netrin‐1 signaling in HSCs.

To provide in vivo evidence that UNC5B mediates Netrin‐1‐induced HSC activation, we injected mice with a lentivirus carrying Unc5b‐shRNA, followed by HFMCD feeding. While no significant differences in body or liver weight were observed, plasma ALT and AST levels were significantly lower in Unc5b‐knockdown mice than in controls (Figure 6H–K). Histological analysis, including Sirius Red staining and Masson's trichrome staining, along with hydroxyproline quantification, revealed markedly attenuated liver fibrosis in Unc5b‐knockdown mice (Figure 6M,N). Consistently, qPCR analysis showed reduced expression of fibrosis‐related genes (Col1a1, Col1a2, and Col3a1) and inflammation‐related genes (Ccl2 and Tnfa) in Unc5b‐knockdown livers (Figure 6O). Furthermore, the levels of Collagen I, α‐SMA, phosphorylated CaMKII, and phosphorylated SMAD2 were also decreased in Unc5b‐knockdown livers (Figure 6P). Together, these results offer strong in vivo evidence that Netrin‐1 exerts its pro‐fibrotic effects primarily through UNC5B‐dependent activation of HSCs.

### 2.8 Nanoparticle‐Mediated Inhibition of Netrin‐1 Mitigates Liver Fibrosis

**FIGURE 7:**
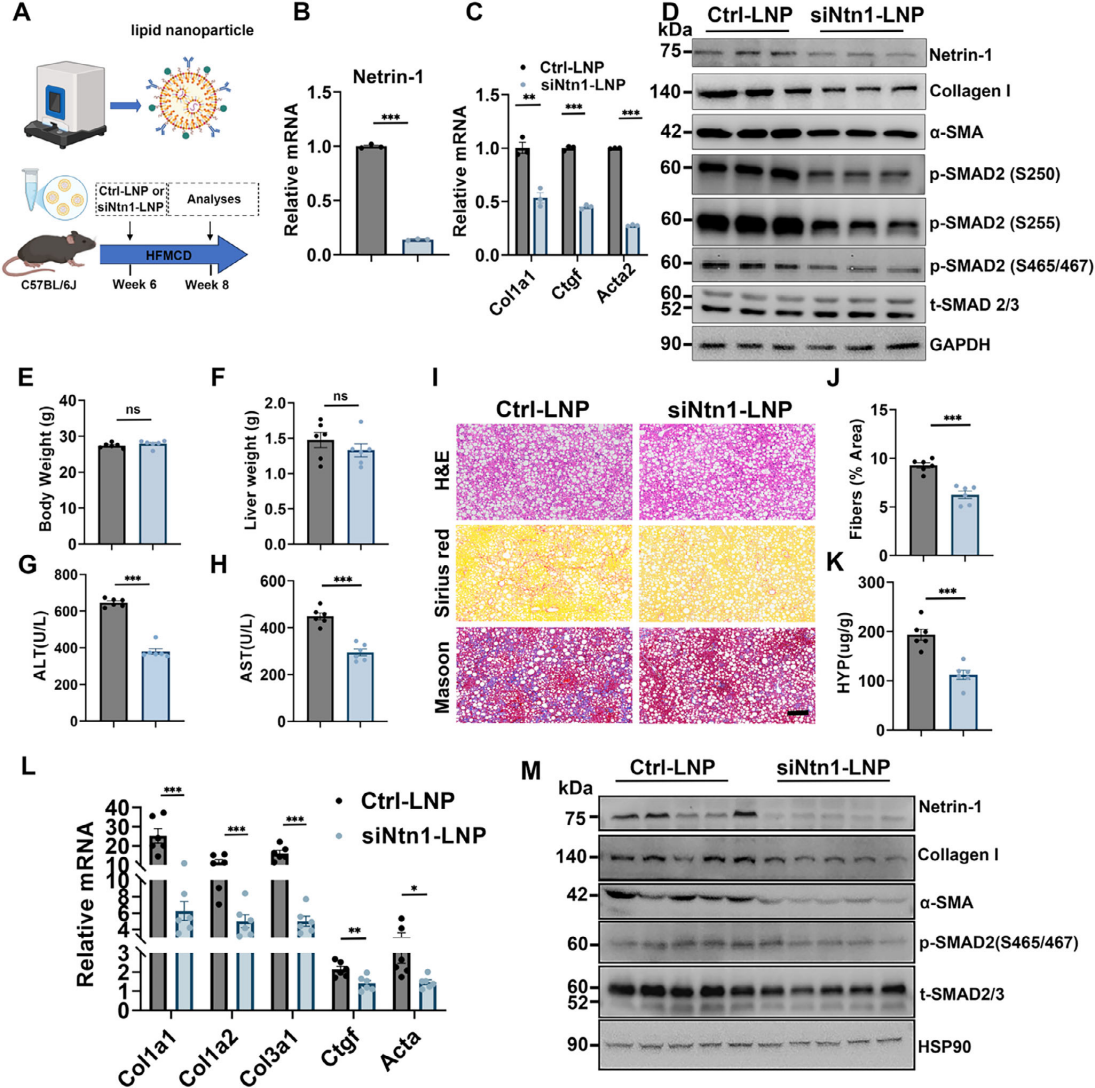
LNP‐mediated RNAi knockdown of Netrin‐1 mitigates liver fibrosis. (A) Schematic diagram of LNP production and experimental design. (B, C) qPCR analysis of gene expression in mHSCs treated with vehicle or siNtn1‐LNP for 24 hrs. (D) Immunoblotting of total cell lysates. (E–M) Mice were fed with HFMCD for 12 weeks and administered with Ctrl‐LNP (n = 6) or siNtn1‐LNP (n=6) via tail vein twice a week for 2 weeks. (E, F) Body and liver weight of treated mice. (G, H) Plasma ALT and AST level. (I) H&E (top), Sirius Red (middle), and Masson's trichrome staining (bottom) of liver sections (scale bar = 100 µm). (J) Quantification of Sirius Red‐positive area in liver sections. (K) Hydroxyproline content in liver tissue. (L) qPCR analysis of hepatic gene expressions. (M) Immunoblotting of total liver lysates. Data are presented as mean ± SEM. * p < 0.05, ** p < 0.01, ***p < 0.001, two‐tailed unpaired Student's *t*‐test.

To directly evaluate the therapeutic potential of targeting the Netrin‐1 pathway in liver fibrosis, we developed lipid‐based nanoparticles (LNP) for siRNA delivery into HSCs (Figure 7A). The physicochemical properties of the prepared siRNA‐LNPs were characterized (Figure S9A–C). We then tested the efficacy of siNtn1‐LNP in cultured mHSCs. Compared with control, siNtn1‐LNP significantly reduced Netrin‐1 mRNA and protein levels (Figure 7B). LNP‐mediated Netrin‐1 knockdown also decreased the mRNA expression of Col1a1, Ctgf, and Acta2 (Figure 7C). Correspondingly, protein levels of collagen I and α‐SMA were also reduced after siNtn1‐LNP treatments (Figure 7D).

We administered control or siNtn1‐LNP to mice fed the HFMCD diet for 12 weeks. Compared with control, siNtn1‐LNP treatment significantly reduced Netrin‐1 protein expression in the liver (Figure 7M). Body and liver weight were comparable between groups; however, plasma ALT and AST levels were significantly lower in the siNtn1‐LNP group (Figure 7E–H). Histological analysis showed that siNtn1‐LNP attenuated liver fibrosis in treated mice, as evidenced using Sirius Red and Masson's trichrome staining (Figure 7I,J). Consistent with these findings, liver hydroxyproline content and the levels of Collagen I, α‐SMA, and phosphorylated SMAD2 were reduced in the LNP‐mediated Netrin‐1 knockdown group (Figure 7K–M). We further confirmed the efficacy of siNtn1‐LNP in CCl4‐induced liver injury and fibrosis models, with results consistent with our previous findings (Figure S9D–H). Together, these data demonstrate that targeting the Netrin‐1 pathway is a feasible strategy to mitigate liver fibrosis.

## 3 Discussion

**FIGURE 8:**
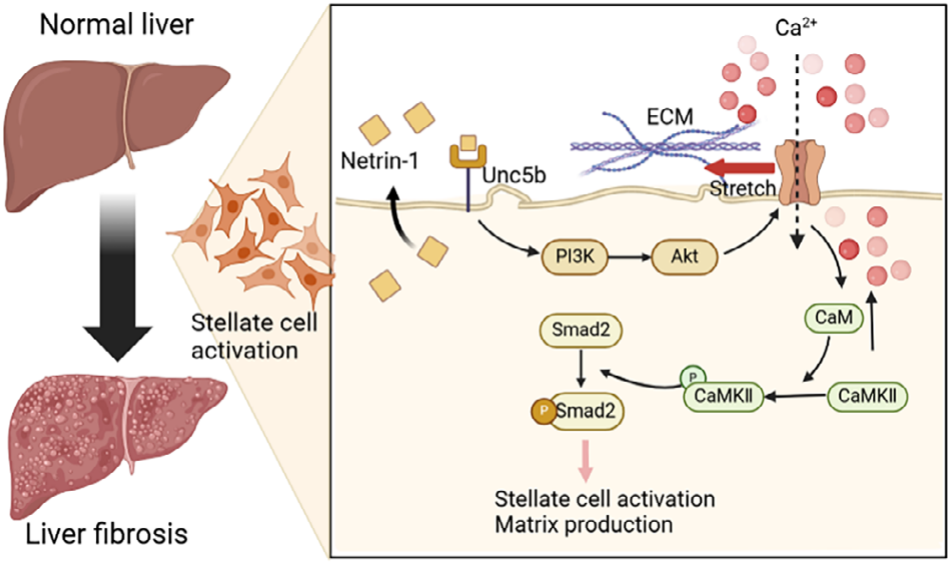
Autocrine Netrin‐1 signaling promotes HSC activation and liver fibrosis. Netrin‐1 expression is upregulated in HSCs during metabolic dysfunction–associated steatohepatitis and injury‐mediated liver fibrosis. Secreted Netrin‐1 establishes an autocrine positive feedback loop by binding to UNC5B receptors on HSCs. Receptor activation triggers calcium influx and profibrotic response in HSCs, leading to increased ECM production and liver fibrosis.

Liver fibrosis is a common pathological feature of chronic liver disease. Advanced fibrosis disrupts normal liver function and increases the risk of end‐stage liver diseases, including cirrhosis and liver cancer [44]. Activated HSCs are a primary source of ECM proteins that drive collagen deposition in the liver [45], while portal fibroblasts also contribute to ECM production, particularly in cholestatic liver disease [46]. Extracellular factors, including TGF‐β, platelet‐derived growth factor, and cytokines such as IL‐6, are key regulators of several aspects of HSC biology, influencing their activation, proliferation, and survival [47, 48, 49, 50, 51]. However, the role of HSC‐derived secreted factors in the autocrine regulation of HSC activation remains largely unexplored. In this study, we performed single‐cell transcriptomic analysis of liver cells to identify HSC‐enriched ligands and receptors involved in the fibrotic response. We uncovered a novel autocrine signaling module mediated by Netrin‐1 and UNC5B that drives HSC activation and liver fibrosis (Figure 8).

Within the liver, Netrin‐1 is most abundantly expressed by HSCs and a subset of liver endothelial cells. Immunofluorescence staining of liver sections showed that Netrin‐1 protein is primarily colocalized with α‐SMA, a marker of activated HSCs. Conversely, a previous study demonstrated that macrophages are the main source of Netrin‐1 in lung fibrosis [19]; however, we did not observe co‐localization of Netrin‐1 with F4/80, a macrophage marker. These findings suggest that the cellular sources contributing to Netrin‐1‐mediated fibrosis may be tissue context‐dependent. Notably, Netrin‐1 expression in the liver is strongly induced during liver fibrosis. Hepatic Netrin‐1 mRNA and protein levels were significantly elevated in diet‐induced MASH (HFMCD) and following CCl4 treatment. This induction coincided with HSC activation and the fibrotic response in these liver injury models, suggesting that Netrin‐1 directly contributes to liver fibrosis progression under these pathological conditions. Supporting this, AAV‐mediated and mRNA‐LNP‐mediated (Figure S10) overexpression of Netrin‐1 exacerbated the severity of liver fibrosis in experimental models, whereas HSC‐specific ablation of Netrin‐1 attenuated liver fibrosis in diet‐induced MASH and CCl4‐induced liver fibrosis. Importantly, LNP‐mediated siRNA knockdown of Netrin‐1 attenuated liver fibrosis and ameliorated diet‐induced MASH in mice. These gain and loss‐of‐function studies highlight the crucial role of Netrin‐1 signaling in driving the fibrotic response in liver disease.

Netrin‐1 plays a complex role in the regulation of axon guidance through its receptors UNC5B and DCC. While DCC expression is generally low in liver cell types, UNC5B is highly expressed in HSCs, supporting an autocrine mechanism in mediating Netrin‐1 action during liver fibrosis. Recombinant Netrin‐1 enhanced a robust fibrotic response in cultured HSCs in a cell‐autonomous manner, as evidenced by the induction of a panel of genes involved in liver fibrosis. UNC5B knockdown significantly reduced the profibrogenic response to Netrin‐1 treatments in HSCs. Mechanistically, Netrin‐1 triggered a rapid rise in intracellular calcium levels, activating CaMK II. Previous studies have highlighted crosstalk between calcium signaling and TGF‐β signaling in regulating fibrotic responses. In this context, TGF‐β elevates cytosolic Ca^2+^ levels, promoting CaMK II activation and the expression of ECM proteins such as collagen and fibronectin [52]. Interestingly, Netrin‐1 engages the Ca^2+^/CaMKII pathway and stimulates SMAD2 phosphorylation in cultured mHSCs. Together, these findings strongly suggest that Netrin‐1 promotes HSC activation and liver fibrosis in a manner analogous to TGF‐β.

Previous studies have highlighted protective roles of Netrin‐1 in the liver; however, these effects appear highly context‐dependent. Netrin‐1 has been reported to attenuate hepatic steatosis by reducing inflammation and alleviating ER stress through the UNC5B/PPARγ pathway [53], and to protect against acetaminophen‐induced hepatotoxicity [54]. These benefits were observed predominantly under metabolic stress or acute liver injury. Conversely, our study focused on chronic fibrotic progression during MASH, a pathological stage characterized by persistent inflammation, extracellular matrix remodeling, and sustained HSC activation. In this context, we demonstrated that HSC‐derived Netrin‐1 engages an autocrine Netrin‐1–UNC5B signaling axis to drive HSC activation and promote liver fibrosis.

These findings indicate that Netrin‐1 may exert dual or opposing functions depending on the disease stage, cellular source, and microenvironmental cues: protective during early metabolic stress or acute injury, but profibrotic during chronic inflammation and tissue remodeling. This duality highlights the importance of considering the temporal and cell‐type–specific actions of Netrin‐1 when evaluating the physiological roles or therapeutic potential. Future studies dissecting these context‐dependent mechanisms will be critical to understanding how Netrin‐1 shifts from a protective mediator to a driver of fibrogenesis in chronic liver disease.

## 4 Experimental Section

### 4.1 Animal Studies

All animal studies were conducted after procedures approved by the Institutional Animal Care and Use Committee of Fudan University (Approval No. ZS112 and DSF‐2021‐016). Mice were housed in pathogen‐free facilities under 12‐h light‐dark cycles with free access to food and water.

To induce MASH, C57BL/6J mice were fed an HFMCD diet (L‐amino acid diet with 45 kcal% fat, 0.1% methionine, and no added choline; A06071309, Research Diets Inc.) for 12 weeks, or a GAN diet (40 kcal% fat, primarily palm oil; 20 kcal% fructose; 2% cholesterol; D09100310, Research Diets Inc.) for 5 months. For diet‐induced metabolic dysfunction‐associated fatty liver disease, mice were fed an HFD (60 kcal% fat, D12492, Research Diets Inc.) for the indicated durations. To induce liver injury and fibrosis, CCl4 was diluted 1:6 in olive oil and injected intraperitoneally at 0.6 ml/kg body weight twice per week for 3 weeks.

To induce cholestasis‐related liver fibrosis, 8‐week‐old C57BL/6 mice were fed chow containing 0.1% DDC (Sigma‐Aldrich, 137030‐25G) for 1 month. Moreover, the BDL‐induced fibrosis model was used, kindly provided by Prof. Yueguo Li (Tianjin Medical University Cancer Institute and Hospital) [55].

For AAV8 transduction, AAV‐Vector or AAV–Ntn1 (1×10^12 genome copies/mouse) was delivered via tail vein injection following either MASH diet feeding or 2 weeks of CCl4 treatment.

For Unc5b knockdown, 8‐week‐old C57BL/6 mice were intravenously injected with 1×10^8 TU of lentivirus per mouse via the tail vein, followed by an HFMCD feeding.

Ntn1‐flox mice (strain no. T036691) and Lrat‐P2A‐iCre mice (strain no. T006205) were obtained from Gem Pharmatech (Nanjing, China). LoxP sites were inserted flanking exons 1‐3 of Ntn1 (NCBI Gene: 18208) in C57B/6 embryonic stem (ES) cells via homologous recombination. Gene‐targeted ES cells were microinjected into mouse blastocysts to generate transgenic germline offspring. Ntn1^fl/fl^ mice were then crossbred with hepatic stellate cell‐specific Lrat‐P2A‐iCre mice line to achieve HSC‐specific Ntn1 knockout.

### 4.2 Plasma Liver Enzymes and Liver Hydroxyproline Content Analysis

Plasma ALT and AST levels were assessed using commercial kits from Nanjing Jianchen Bioengineering Institute (NJJCBIO, C009‐2‐1 and C010‐2‐1) according to the manufacturer's instructions. Liver hydroxyproline content was determined as previously described [56]. Briefly, liver tissue was homogenized in water and hydrolyzed by incubation with 6 N hydrochloric acid at 120°C for 5 h. Hydrolysates were then analyzed using a Hydroxyproline Colorimetric Assay Kit (NJJCBIO, A030‐3‐1).

### 4.3 Histology Analysis

Liver tissues were immediately fixed in 10% formalin at 4°C overnight after removal, then processed for paraffin embedding and H&E staining. Sirius Red and Masson's trichrome staining were conducted as previously described [57]. The percentage of Sirius Red‐positive area relative to the total view area was quantified using ImageJ.

### 4.4 Gene Expression Analysis

qPCR was used to analyze gene expression levels. Briefly, mouse livers were immediately frozen in liquid nitrogen after removal. Total RNA was extracted using TRIzol reagent (Vazyme Biotech, R401‐01), and 1 µg of total RNA was reverse‐transcribed using MMLV‐RT (Vazyme Biotech, R222‐01). qPCR was performed using SYBR Green (Vazyme Biotech, Q311‐02). Relative mRNA abundance was normalized to the internal control gene encoding the ribosomal protein 36B4. The sequences of the qPCR primers are listed in Table S1.

RNA sequencing was performed using the Illumina NovaSeq 6000 platform at GENEWIZ, Inc., Suzhou. Raw sequencing read counts were normalized and processed using DESeq2. Significantly expressed genes were identified with a false discovery rate of less than 0.05. The RNA‐seq data generated in this study have been deposited in the Gene Expression Omnibus database (GSE285431 and GSE303583).

### 4.5 Immunoblotting Analysis

Snap‐frozen liver tissues were homogenized in a lysis buffer containing 50 mM Tris‐HCl (pH 7.5), 137 mM NaCl, 1 mM ethylenediaminetetraacetic acid, 1% Triton X‐100, 10% glycerol, 10 mM NaF, 10 mM Na_4_P_2_O_7_, 1 mM Na_3_VO_4_, and an inhibitor cocktail. Cell cultures were washed twice with ice‐cold PBS and then lysed in RIPA buffer containing inhibitors. Lysates were separated by sodium dodecyl sulfate‐polyacrylamide gel electrophoresis, transferred to polyvinylidene fluoride membranes, and then immunoblotted with primary and secondary antibodies. The following antibodies were used: anti‐α‐SMA, Santa Cruz BioTech, sc‐53142; anti‐Netrin‐1, Abcam, ab126729; anti‐GAPDH, Proteintech, 60004‐1‐Ig; anti‐Collagen type I, Proteintech, 14695‐1‐AP; anti‐Phospho‐NF‐kappaB p65 (Ser536) (93H1), Cell Signaling Technology, 3033T; anti‐NF‐kappaB p65, Cell Signaling Technology, 8242T; anti‐Phospho‐Smad2 (Ser465/467), Cell Signaling Technology, 3108T; anti‐Phospho‐Smad2 (Ser255), Selleck, F1689; anti‐Phospho‐Smad2 (Ser250), Abmart, T55857; anti‐Smad2/3, Santa Cruz BioTech, sc133098; anti‐HSP90, Proteintech, 13171‐1‐AP; anti‐ Phospho‐SAPK/JNK (Thr183/Tyr185), Cell Signaling Technology, 4668T; anti‐JNK, Beyotime BioTech, AF1048; anti‐Phospho‐p44/42 MAPK(Erk1/2)(Thr202/Tyr204), Cell Signaling Technology, 4370T; anti‐p44/42 MAPK(Erk1/2), Cell Signaling Technology, 4695T; anti‐CaMKII, Affinit, DF2907; anti‐Phospho‐CaMKII; Affinit; AF3493; anti‐UNC5H2, R&D system, AF1006; Peroxidase‐AffiniPure Goat Anti‐Mouse IgG (H+L), Jackson, 115‐035‐003; Peroxidase‐AffiniPure Goat Anti‐Rabbit IgG (H+L), Jackson, 111‐035‐003.

### 4.6 Immunofluorescence Staining

Liver tissues were fixed in 4% paraformaldehyde for 4 hrs and embedded in optimal cutting temperature compound. For mHSCs, cells that were cultured on glass coverslips underwent fixation in 4% paraformaldehyde for 30 min. Frozen liver sections or cells were permeabilized with 0.2% Triton X‐100 in PBS for 15 min and blocked with 5% bovine serum albumin for 1 h, followed by incubation with primary antibody solution overnight at 4°C and secondary antibody solution at room temperature for 1 h.

Sections were mounted using ProLong Gold Antifade Mountant (Thermo Fisher Scientific, P10144). Images were captured using a fluorescence microscope (OLYMPUS VS200). Immunofluorescence experiments were conducted using the following antibodies: anti‐α‐SMA (Proteintech, 14395‐1‐AP), anti‐Netrin‐1 (R&D System, AF1109‐SP), anti‐F4/80 (Thermo Fisher Scientific, 14‐4801‐82), anti‐Vimentin, Proteintech, 10366‐1‐AP, CoraLite488‐conjugated Goat Anti‐Rabbit IgG(H+L) (Proteintech, SA00013‐2), Donkey Anti‐Goat IgG H&L (Cy5) (Abcam, ab6566), Donkey anti‐Rat IgG (H+L) Highly Cross‐Adsorbed Secondary Antibody, Alexa Fluor 488 (Thermo Fisher Scientific, A‐20218).

### 4.7 Hepatic Stellate Cell Isolation, Culture, Immortalization, and Treatment

Primary hepatic stellate cells (pHSCs) were isolated from mouse livers using pronase/collagenase digestion followed by gradient centrifugation, as previously described [12]. pHSCs were specifically used in Figure 1D, Figure 6C and Figure S4L&M. In these figures, protein and RNA analyses were carried out immediately after pHSC isolation.

All in vitro treatments described in the study were performed using immortalized mouse HSCs (mHSCs). Isolated pHSCs were cultured in Dulbecco's Modified Eagle Medium (DMEM) supplemented with 10% fetal bovine serum (FBS). Cultured pHSCs were infected with an SV40 large T antigen‐expressing retrovirus and selected with G418 solution. Immortalized mouse HSCs were maintained in DMEM medium with 10% FBS at 37°C under 5% CO_2_. Prior to treatments, cells were switched to serum‐free DMEM for 12 h. Treatments included Netrin‐1 (R&D System, 1109‐N1‐025) and KN93 (MCE, HY‐15465). No TGF‐β was added prior to the experimental treatments.

### 4.8 Calcium Imaging

The mHSCs were seeded onto cover glasses and cultured for 48 hrs. Cells were loaded with the calcium indicator FURA‐2AM (Beyotime BioTech, s1052) and imaged using an inverted fluorescence microscope. Images were acquired every other second to monitor dynamic changes in intracellular calcium levels. The mHSCs were treated with 300 ng/ml recombinant Netrin‐1 protein (R&D System, 1109‐N1‐025) or 10 ng/ml ang II (Selleck, P1085) in the presence of an indicator.

### 4.9 Pharmacological Ablation of Sympathetic Nerves

Peripheral sympathetic nerve terminals were chemically ablated by intraperitoneal administration of 6‐OHDA (Sigma, H4381) at 100 mg/kg, twice per week for 2 weeks. The neurotoxin was freshly dissolved in saline supplemented with 0.1% (w/v) ascorbic acid (Beyotime Biotech, Shanghai, China). Control mice received vehicle alone. Successful sympathectomy was confirmed using 3D liver imaging using an anti‐tyrosine hydroxylase antibody (AB152; Sigma–Aldrich).

### 4.10 Lipid‐Based Nanoparticles Formulation and Characterization

Formulation was performed as previously described [58, 59]. An ethanol‐based organic phase containing the ionizable lipid AA‐T3A (MedChemExpress, HY‐148859), distearoylphosphatidylcholine (Avanti Polar Lipids, 850365), cholesterol (Avanti Polar Lipids, 700100), and DMPE‐PEG2000 (Avanti Polar Lipids, 880150) at a molar ratio of 50:10:38.5:1.5 was prepared. Separately, an aqueous phase containing siNtn1 was prepared in 10 mM citrate buffer (pH 3.0). LNPs were formed by mixing the aqueous and organic phases using a staggered herringbone micromixer microfluidic chip. The flow rates were set to 1.8 mL/min for the aqueous phase and 0.6 mL/min for the organic phase, achieving a 3:1 aqueous‐to‐organic volume ratio. Mixing was performed using 33 DDS syringe pumps (Fudan University, Shanghai, China). The resulting LNPs were characterized for hydrodynamic diameter, polydispersity index, and zeta potential by dynamic light scattering using a Zetasizer Nano ZS90 instrument (Malvern Panalytical, UK; located at Fudan University, Shanghai, China).

### 4.11 Netrin‐1 Ablation with Lipid Nanoparticles

The mHSCs were seeded on coverslips and cultured for 24 hrs before treatment with 10 nM siRNA equivalent of AA‐T3A‐C12/siGFP LNPs or AA‐T3A‐C12/siNtn1 LNPs for 48 h. For in vivo studies, liver fibrosis was induced in C57BL/6J mice either by intraperitoneal injection of CCl4 or by feeding an HFMCD diet. During the final 2 weeks of fibrotic induction, mice received biweekly intravenous injections of AA‐T3A‐C12/siGFP LNPs or AA‐T3A‐C12/siNtn1 LNPs at a fixed siRNA dose of 25 µg per injection (equivalent to 1 mg/kg body weight).

### 4.12 Statistical Analyses and Data Visualization

Statistical analyses were performed using GraphPad Prism 10. Differences were assessed using a two‐tailed unpaired Student's *t*‐test. A p‐value of less than 0.05 (*p < 0.05; **p < 0.01) was considered statistically significant. UMAP plots, violin plots, feature plots, and heat maps were generated using R.

## Author Contributions

Jiahui Zhao, Bo Wang, and Xuelian Xiong conceived the study. Jiahui Zhao, Yajie Peng, Tianyi Wang, and Huajuan Wang conducted the experiments. Hongyan Lei assisted with RNA sequencing data analysis and visualization. Jiahui Zhao, Yajie Peng, and Hongyan Lei contributed equally to this study.

## Funding

This work was supported by grants from the National Key Research and Development Program of China (2021YFA0805500 and 2020YFA0803604).

## Conflicts of Interest

The authors declare no conflicts of interest.

## Supporting information




**Supporting File**: advs73721‐sup‐0001‐SuppMat.docx.

## Data Availability

All data generated in this study are available from the corresponding author upon reasonable request.
