# Supplementary material for: Autocrine Netrin‐1 Signaling in Hepatic Stellate Cells Drives Liver Fibrosis and Diet‐Induced Metabolic Dysfunction‐Associated Steatohepatitis in Mice
Source: Adv Sci (Weinh). 2026 Jan 9;13(10):e14545. doi: 10.1002/advs.202514545 (PMC12915089; doi:10.1002/advs.202514545)
Supplement: Supplementary file 1 — Supporting File: advs73721‐sup‐0001‐SuppMat.docx. [file ADVS-13-e14545-s001.docx]

**
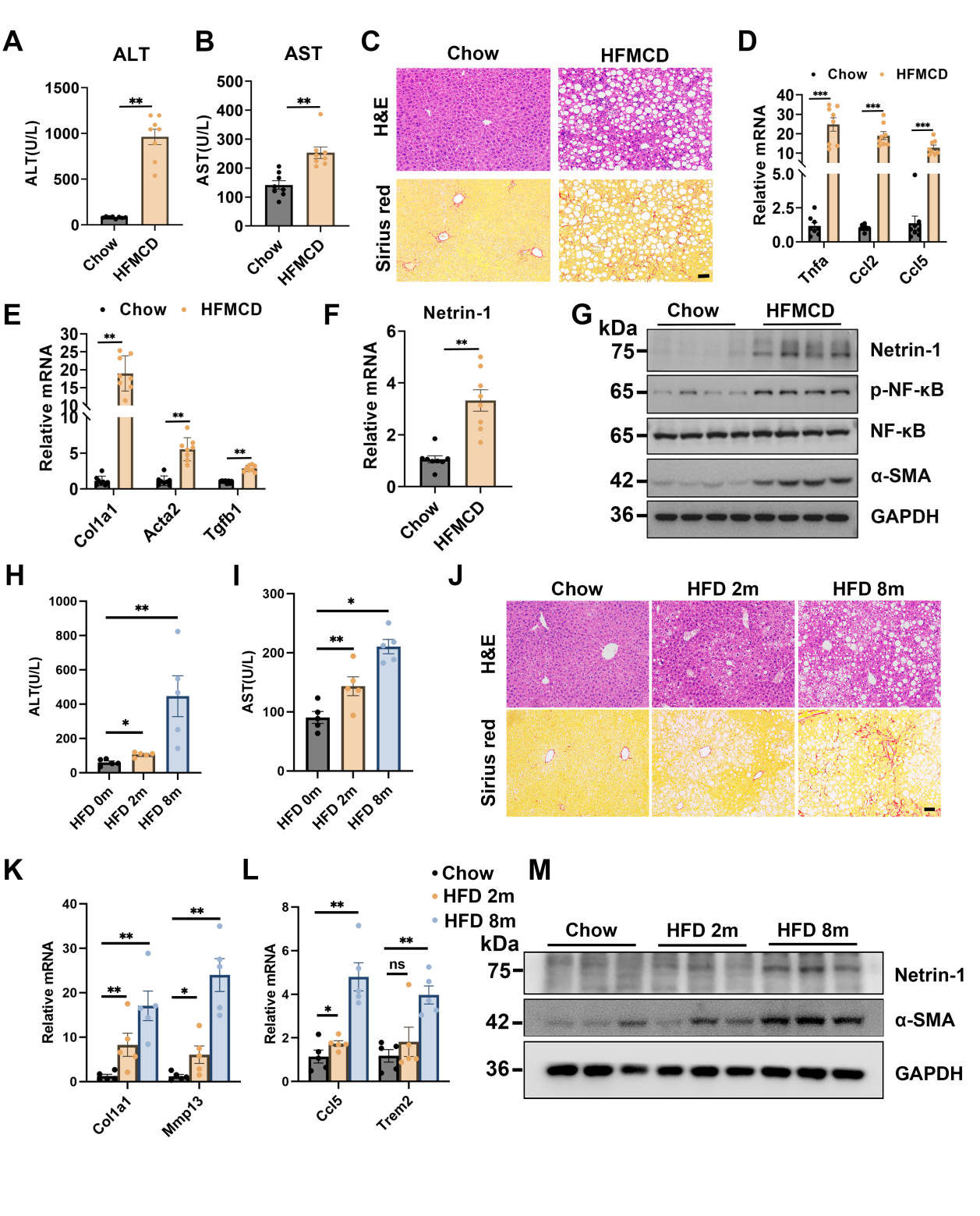
**

**Supplemental Figure 1. Increased Netrin-1 expression in different MASH models**

Mice were fed on chow (n=8) or HFMCD (n=8) diet for 8 weeks. (A-B) Serum ALT and AST level. (C) H&E (top) and Sirius Red (bottom) staining of liver sections (scale bar=100 μm). (D-F) qPCR analysis of hepatic gene expressions. (G) Immunoblotting of total liver lysates. For the high fat diet, mice were fed on chow diet or high fat diet for 2 months (HFD 2m, n=5) and 8 months (HFD 8m, n=5) respectively. (H, I) Serum ALT and AST levels. (J) H&E (top) and Sirius Red (bottom) staining of liver sections (scale bar=100 μm). (K-L) qPCR analysis of hepatic gene expressions. (M) Immunoblotting of total liver lysates. Data are presented as the means ± SEM. * p<0.05, ** p<0.01, ***p<0.001, two-tailed unpaired Student's t test.

**
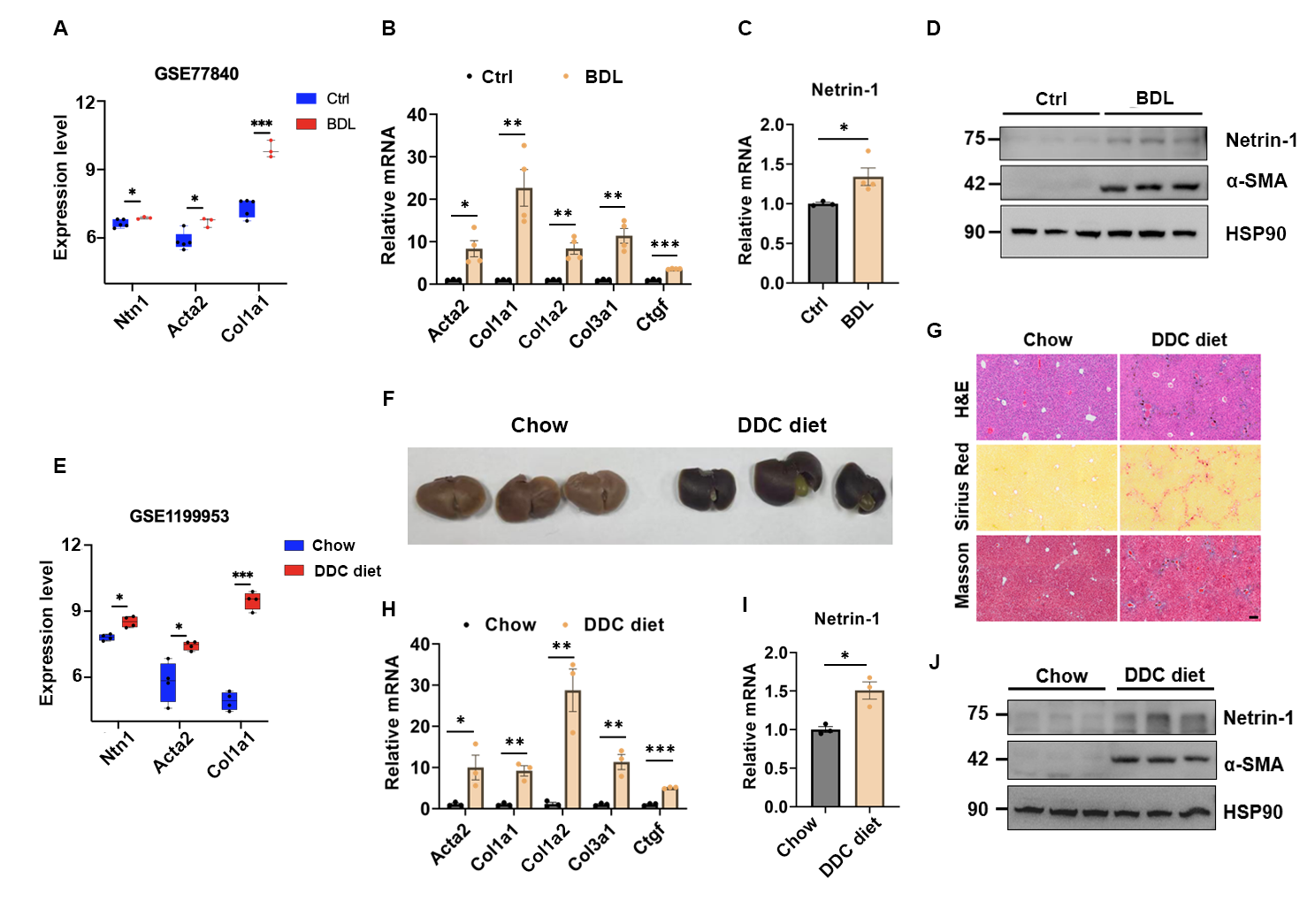
**

**Supplemental Figure 2. Hepatic Netrin-1 expression in BDL and DDC animal models.**

(A) Expression levels of Ntn1, Acta2, and Col1a1 in liver tissues from control (Ctrl) and BDL groups in public databases. GSE77840. (B-D) 8-week-old C57BL/6 mice were subjected to sham operation (Ctrl, n=3) or bile duct ligation (BDL, n=4), and liver samples were collected 2 weeks post-surgery. (B, C) qPCR analysis of hepatic gene expressions. (D) Immunoblotting analysis of Netrin-1 and α-SMA. (E) Expression levels of Ntn1, Acta2, and Col1a1 in liver tissues from control (Ctrl) and DDC-treated groups in public databases. GSE1199953. (F-J) 8-week-old C57BL/6 mice were fed a diet containing 0.1% DDC (Diethyl 1,4-dihydro-2,4,6-trimethyl-3,5-pyridinedicarboxylate, Sigma, 137030-25G) for 1 month (n=3 for each group). (F) Representative gross liver morphology. (G) H&E (top), Sirius Red (middle), and Masson staining (bottom) of liver sections (scale bar=100 μm). (H, I) qPCR analysis of hepatic gene expressions. (J) Immunoblotting analysis of Netrin-1 and α-SMA in mouse livers. Data represent mean ± SEM. *p<0.05, **p<0.01, ***p<0.001; two-tailed unpaired Student's t test.

**
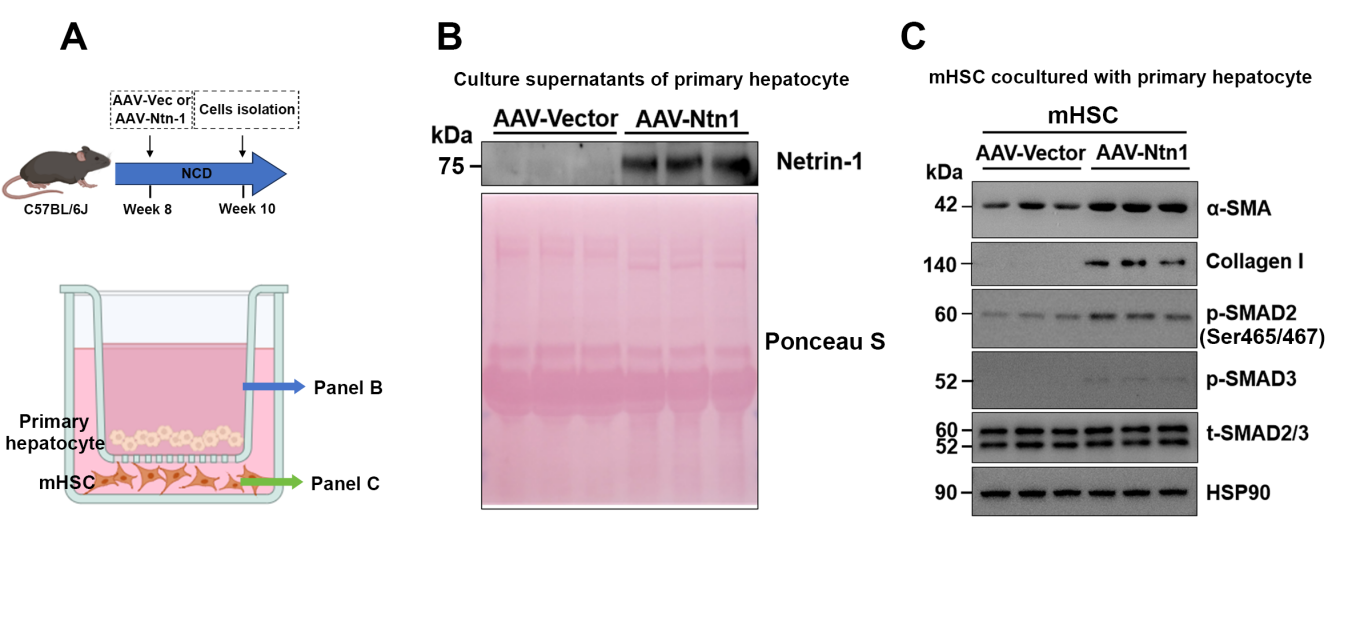
**

**Supplemental Figure 3. Hepatocyte-derived Netrin-1 promotes HSC activation via secreted protein.**

8-week-old C57BL/6 mice were randomly assigned to two groups and received tail vein injections of AAV8-TBG-Vector or AAV8-TBG-Ntn1 (5X10^11 TU per mouse, n=3 per group). Two weeks later, primary hepatocytes were isolated and cultured as illustrated. (A) Experimental design for A-C. (B) Immunoblotting analysis of Netrin-1 in primary hepatocyte culture supernatants collected 48 hrs after plating, with Ponceau S staining shown as loading control. (C) Immunoblotting analysis of fibrosis-related markers in mHSCs co-cultured with conditioned media from hepatocytes.

**
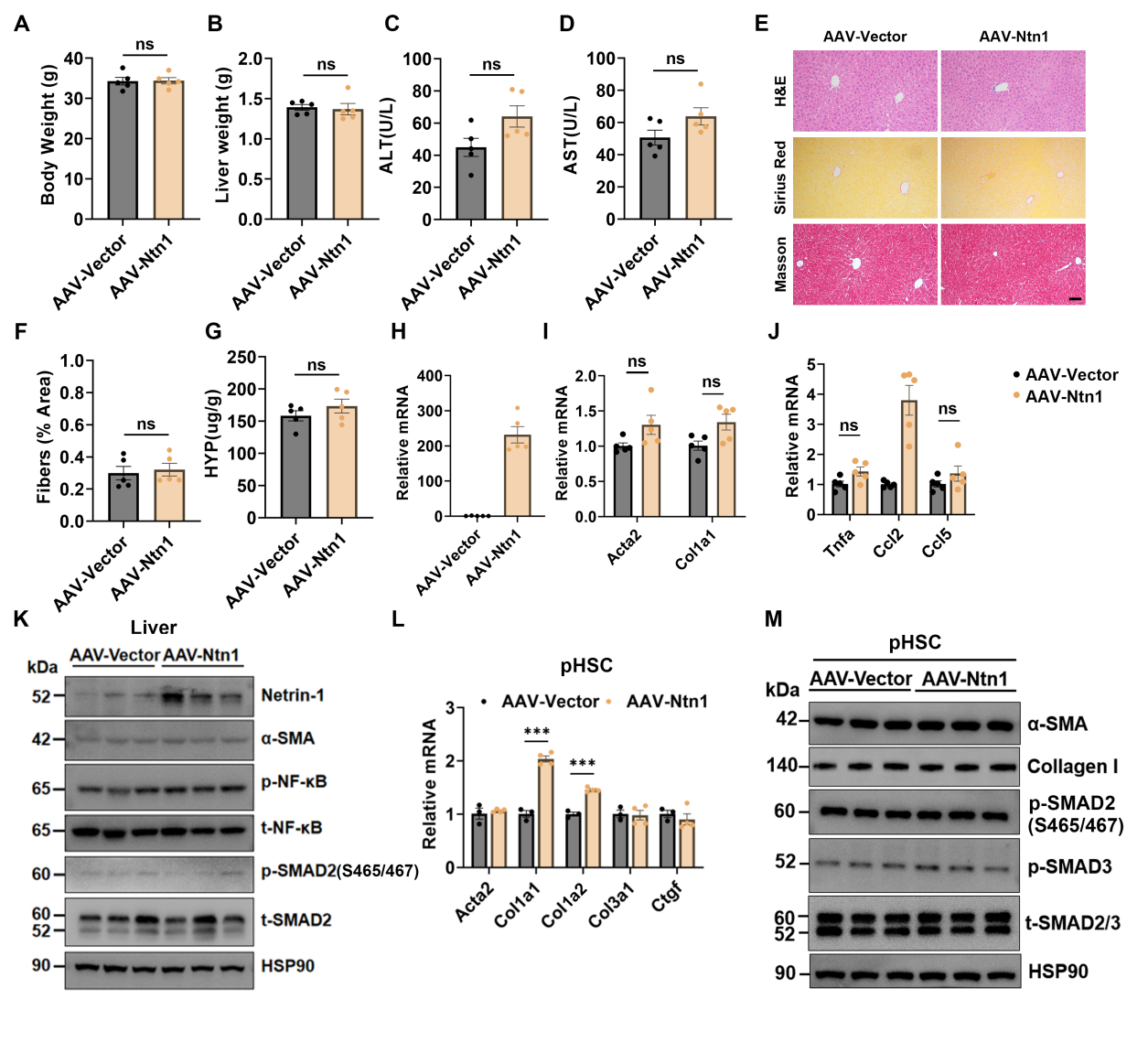
**

**Supplemental Figure 4: Hepatocyte-specific overexpression of Netrin-1 is insufficient to initiate HSC activation or fibrosis.**

8-week-old C57BL/6 mice were randomly assigned to two groups and received tail vein injections of AAV8-TBG-Vector or AAV8-TBG-Ntn1 (5X10^11 TU per mouse). Mice were sacrificed 2 months post-injection for analysis (n=5 for each group).

(A, B) Body weight and liver weight. (C, D) Serum levels of ALT and AST. (E) H&E (top), Sirius Red (middle), and Masson staining (bottom) of liver sections (scale bar=100 μm). (F) Quantification of Sirius Red-positive area on liver sections. (G) Hydroxyproline content of liver tissue. (H–J) qPCR analysis of hepatic Ntn1, fibrosis-related, and inflammation-related genes in liver tissues. (K) Immunoblotting analysis of Netrin-1 and fibrosis-related markers in liver tissues. (L, M) qPCR analysis and immunoblotting analysis of freshly isolated pHSCs. Data represent mean ± SEM. *p<0.05, **p<0.01, ***p<0.001; two-tailed unpaired Student's t test.

**
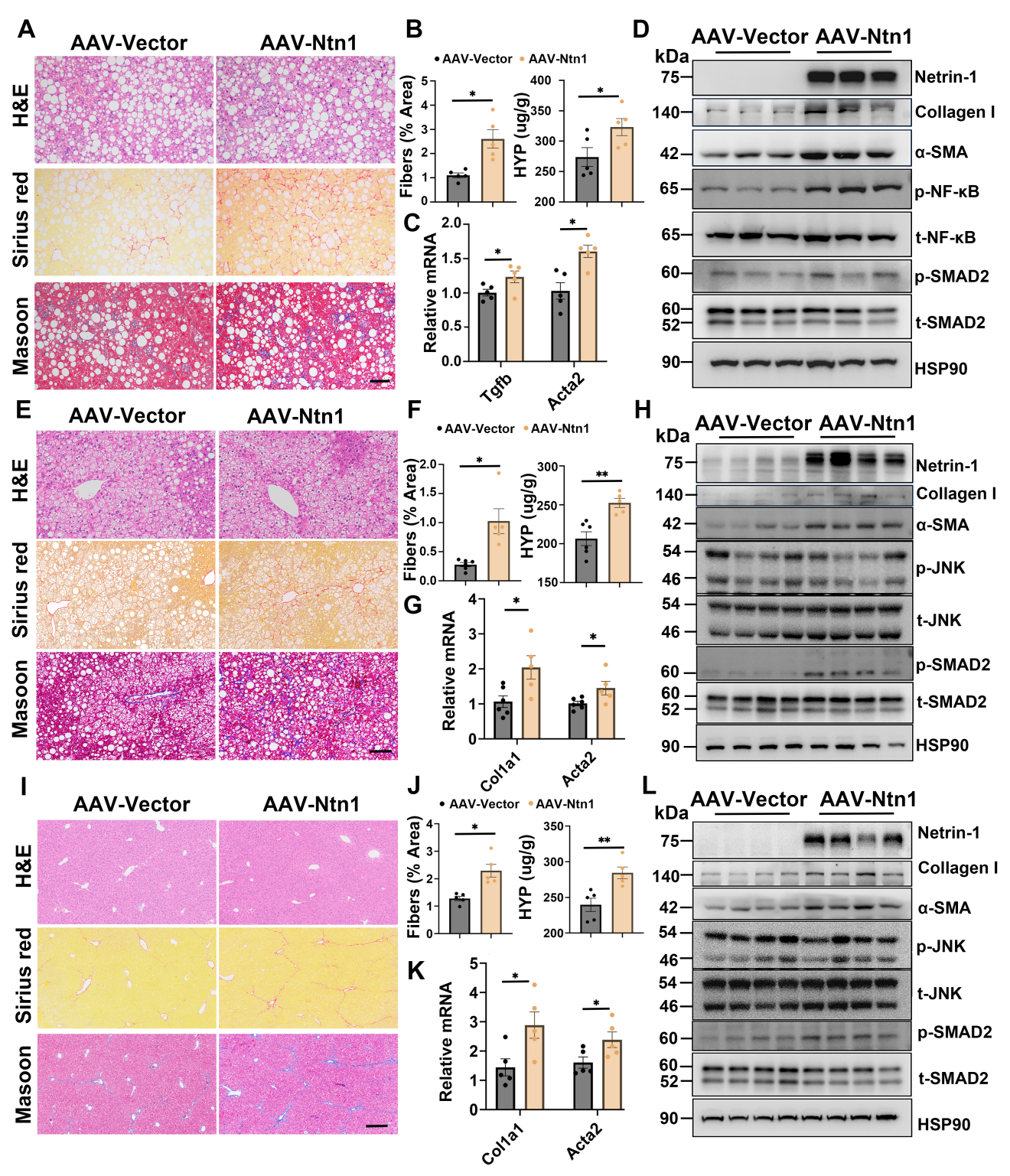
**

**Supplemental Figure 5. Overexpression of Netrin-1 exacerbates liver fibrosis in different MASH models.**

AAV8-TBG-Vector (AAV-Vector, n=5) and AAV8–TBG-Ntn1 (AAV-Ntn1, n=5) mice were fed on HFMCD for 8 weeks. (A) H&E (top), Sirius Red (middle) and Masson staining (bottom) of liver sections (scale bar=100 μm). (B) Quantification of Sirius Red-positive area on liver sections and hydroxyproline content. (C) qPCR analysis of hepatic gene expressions of liver tissue. (D) Immunoblotting of total liver lysates.

AAV-Vector (n=6) and AAV-Ntn1 (n=5) mice were fed on HFD diet for 16 weeks. The following parameters were measured. (E) H&E (top), Sirius Red (middle) and Masson staining (bottom) of liver sections (scale bar=100 μm). (F) Quantification of Sirius Red-positive area on liver sections and hydroxyproline content. (G) qPCR analysis of hepatic gene expressions of liver tissue. (H) Immunoblotting of total liver lysates.

AAV-Vector (n=5) and AAV-Ntn1 (n=5) mice intraperitoneally injected with CCl4 for 2 weeks. (I) H&E (top), Sirius Red (middle) and Masson staining (bottom) of liver sections (scale bar=100 μm). (J) Quantification of Sirius Red-positive area on liver sections and hydroxyproline content. (K) qPCR analysis of hepatic gene expressions of liver tissue. (L) Immunoblotting of total liver lysates. Data represent mean ± SEM. *p<0.05, **p<0.01; two-tailed unpaired Student's t test.


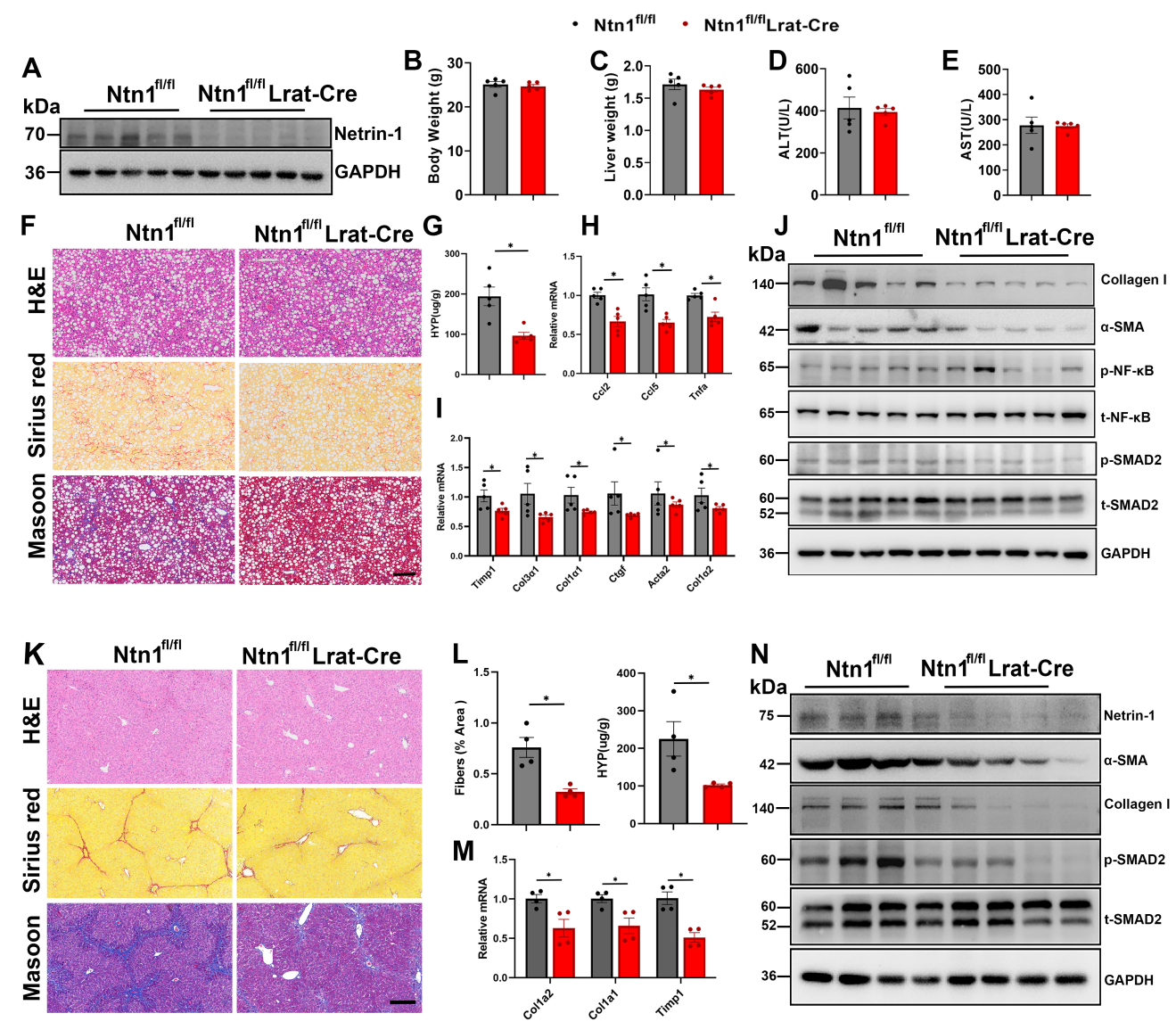


**Supplemental Figure 6. HSC-specific inactivation of Netrin-1 mitigates liver fibrosis in different fibrosis models.**

Ntn1^fl/fl^ (n=5) and Ntn1^fl/fl^Lrat-Cre (n=5) mice were fed on HFMCD diet for 12 weeks. (A) Immunoblotting of total liver lysates. (B, C) Body weight and liver weight of mice. (D, E) Plasma ALT and AST concentrations. (F) H&E (top), Sirius Red (middle) and Masson staining (bottom) of liver sections (scale bar=100 μm). (G) Hydroxyproline content of liver. (H, I) qPCR analysis of hepatic gene expressions. (J) Immunoblotting of total liver lysates.

Ntn1^fl/fl^ (n=4) and Ntn1^fl/fl^Lrat-Cre (n=4) treated with CCl4 twice per week for 3 weeks. (K) H&E (top), Sirius Red (middle) and Masson staining (bottom) of liver sections (scale bar=100 μm). (L) Quantification of Sirius Red-positive area on liver sections and hydroxyproline content of liver. (M, N) qPCR analysis and Immunoblotting of total liver. Data represent mean ± SEM. *p<0.05, **p<0.01; two-tailed unpaired Student's t test.

**
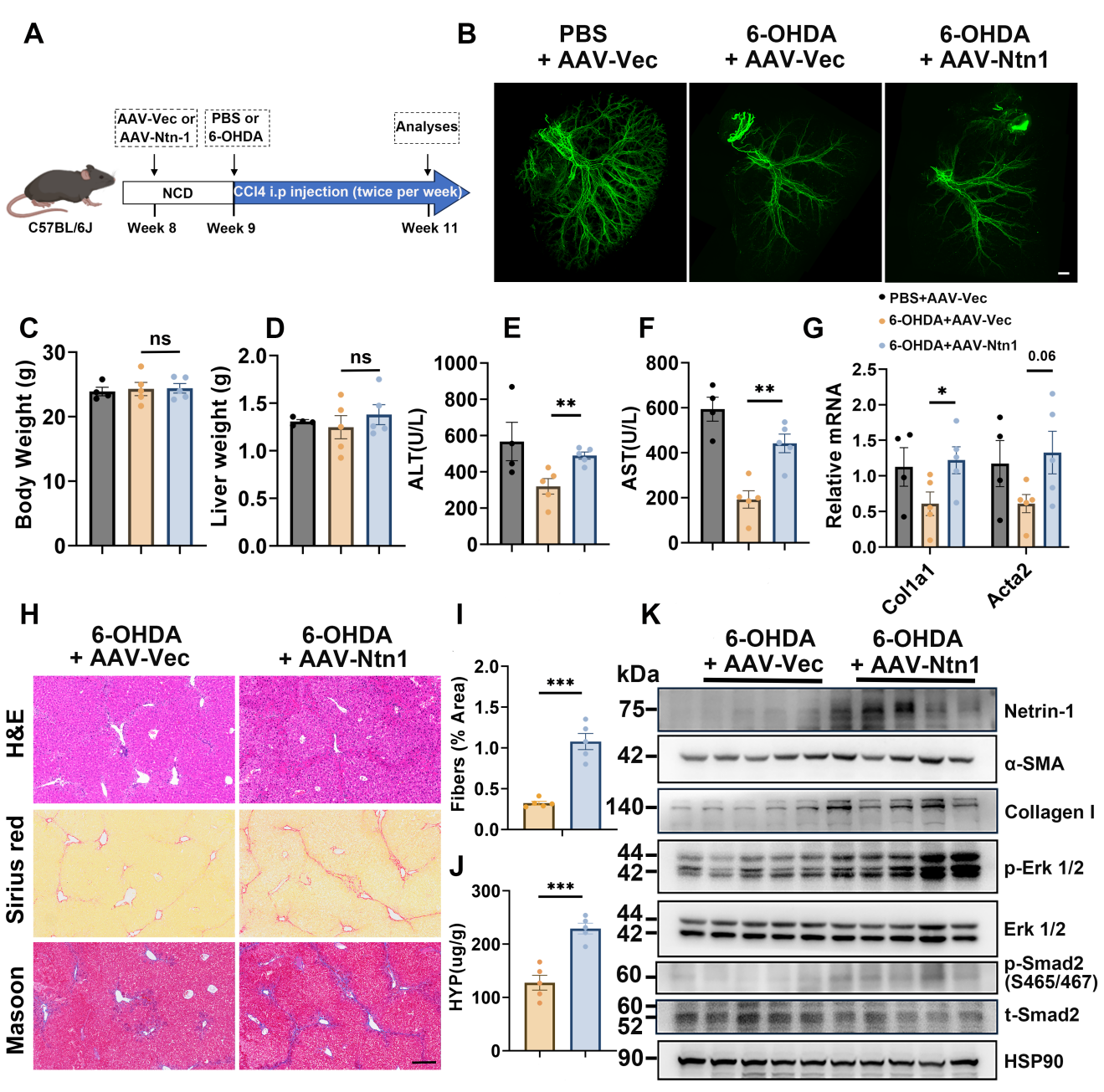
Supplemental Figure 7. Netrin-1 exacerbates liver fibrosis independently of sympathetic innervation.**

1. Schematic diagram of the experimental design. (B) Whole-mount three-dimensional imaging of hepatic sympathetic fibers. (C, D) Body and liver weight. (E, F) Plasma ALT and AST levels. (G) qPCR analysis of hepatic gene expression. (H) H & E (top), Sirius Red (middle), and Masson trichrome staining (bottom) of liver sections (scale bar=100 μm). (I) Quantification of Sirius Red-positive area on liver sections. (J) Liver hydroxyproline content. (K) Immunoblotting of total liver lysates. Data are presented as mean ± SEM. *p<0.05, **p<0.01; two-tailed unpaired Student's t-test.


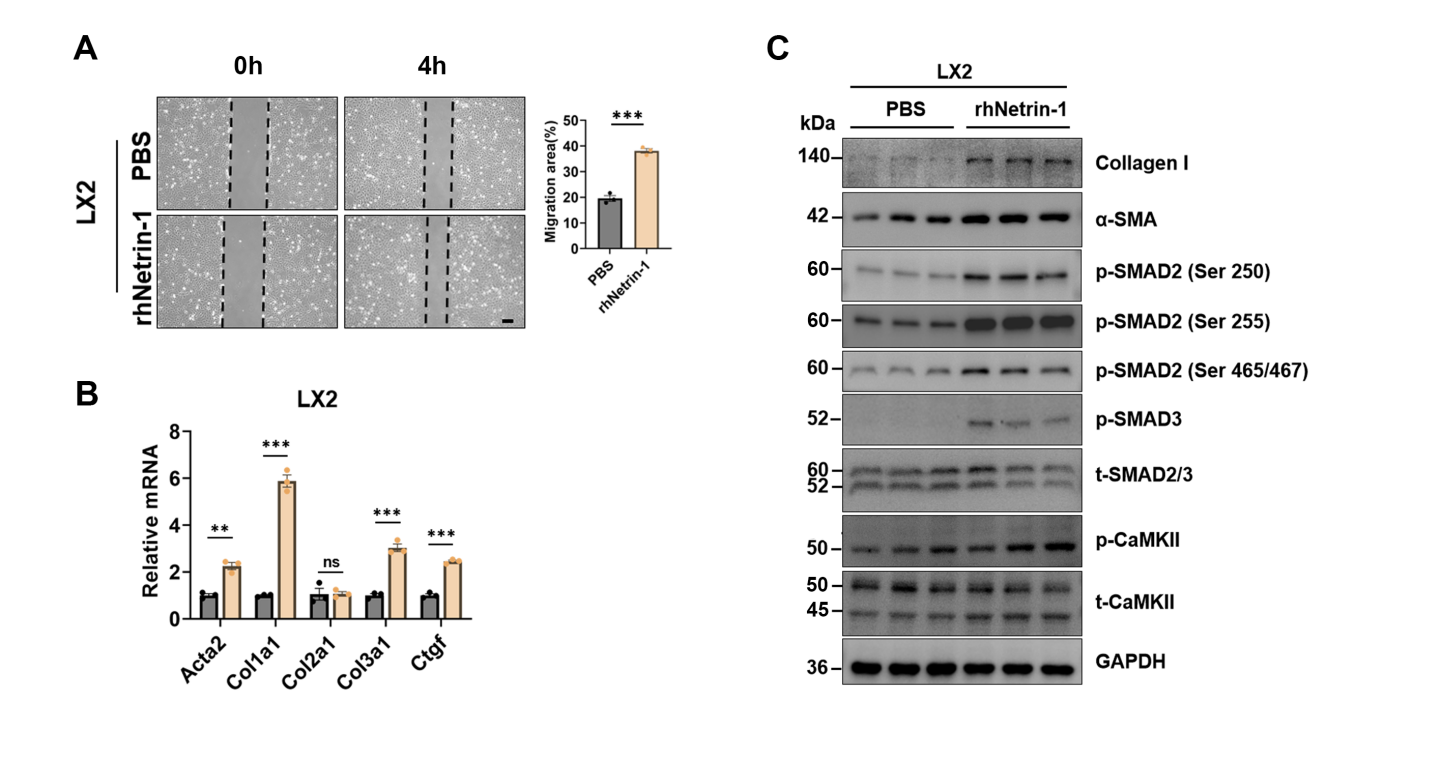


**Supplemental Figure 8. Netrin-1 exerted pro-fibrotic effects on human HSCs in a cell-autonomous manner.**

(A) Wound-healing assay of LX2 cells following rhNetrin-1 (300 ng/ml) treatment. Quantification of migration rate is shown on the right. (B, C) qPCR and Western blot analysis of fibrosis-related genes and proteins in LX2 cells treated with PBS or rhNetrin-1. Data are presented as mean ± SEM; n = 3 biological replicates;*p<0.05, **p<0.01, ***p<0.001; two-tailed unpaired Student's t test.


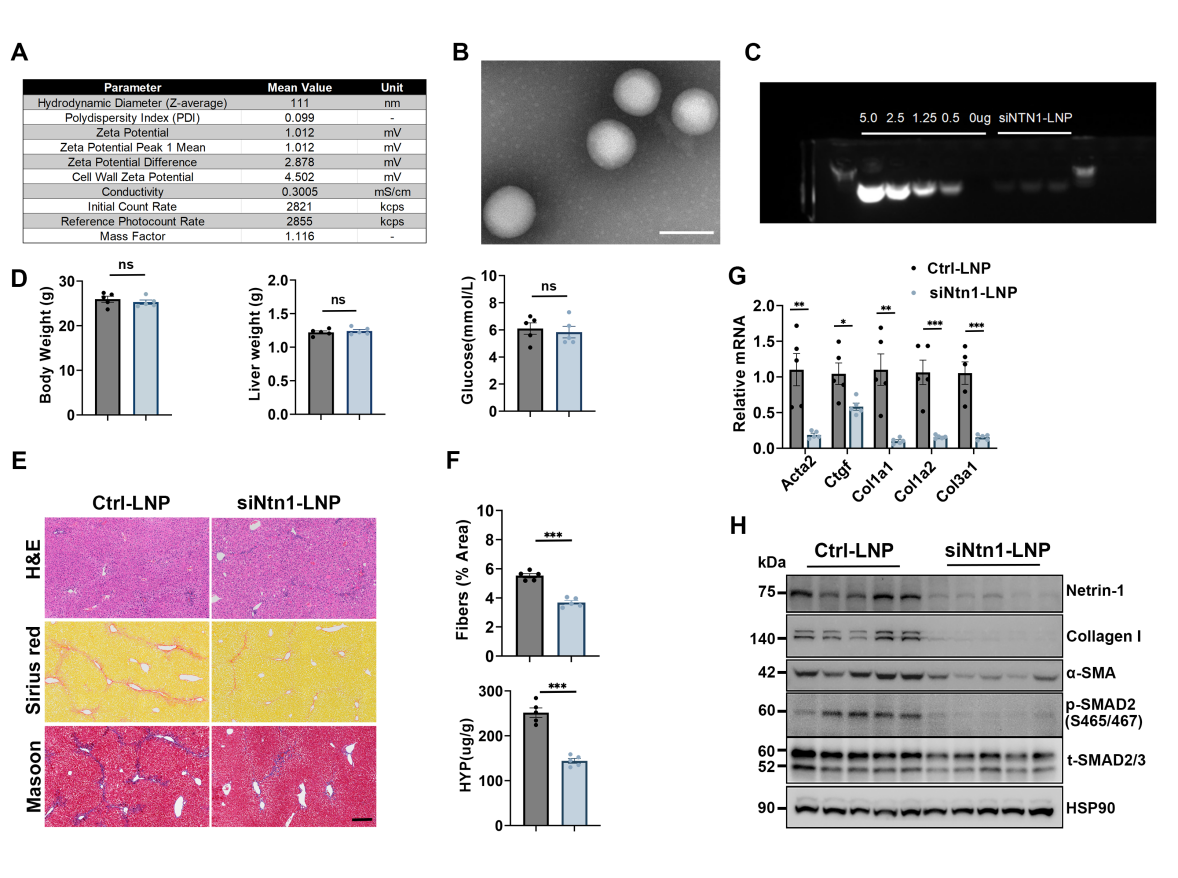


**Supplemental Figure 9. Nanoparticle HSC-target delivery of Ntn1 siRNA attenuates CCl4-induced fibrosis model.**

(A)Characterization of LNP. (B) A representative TEM image of LNP. Scale bar, 100 nm. (C) siRNA with different total amount was analyzed by 1% agarose gel electrophoresis. 5 ug siNtn1-LNP was loaded into the last lane to detect encapsulation efficiency. (D-H) To induce liver injury, mice were injected intraperitoneally with CCl4 (twice per week for 3 weeks). siNtn1-LNPs and vehicle were co-administered by tail vein on the same schedule. n=5 for each group. (D) Body weight, liver weight and glucose of mice. (E) H&E (top), Sirius Red (middle) and Masson staining (bottom) of liver sections (scale bar=100 μm). (F) Quantification of Sirius Red-positive area on liver sections and hydroxyproline content of liver. (G-H) Immunoblotting and qPCR analysis of total liver. Data represent mean ± SEM. *p<0.05, **p<0.01, ***p<0.001; two-tailed unpaired Student's t test.


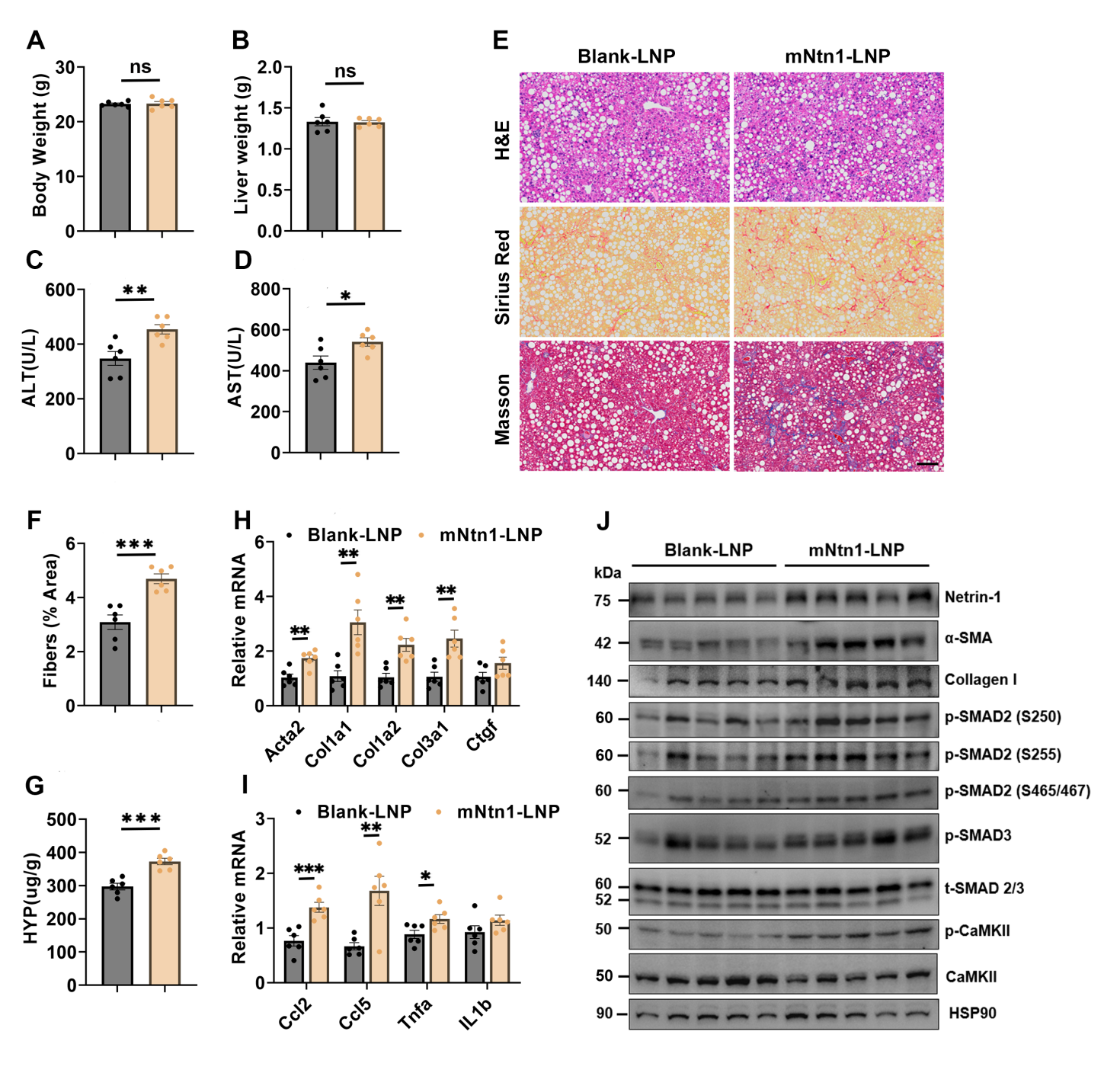


**Supplemental Figure 10. LNP-encapsulated mNtn1 exhibited pro-fibrotic effect in MASH model.**

8-week-old C57BL/6 mice were fed with HFMCD for 2 months. During the last 3 weeks, mice were randomly assigned to two groups (n=6 for each group) and received tail vein injections of vehicle or mNtn1-LNP (20ug per mouse). (A-B) Body weight and liver weight. (C-D) Serum levels of ALT and AST. (E) H&E (top), Sirius Red (middle), and Masson staining (bottom) of liver sections (scale bar=100 μm). (F-G) Quantification of Sirius Red-positive area on liver sections and hydroxyproline content of liver tissue. (H-I) qPCR analysis of fibrosis-related, and inflammation-related genes in liver tissues. (J) Immunoblotting analysis of Netrin-1 and fibrosis-related markers in liver tissues. Data represent mean ± SEM. *p<0.05, **p<0.01, ***p<0.001; two-tailed unpaired Student's t test.

Table S1. Primers used in this study

| Primer | Species | Forward Seq | Reverse Seq |
| --- | --- | --- | --- |
| Tnfa | Mus | AGCCCCCAGTCTGTATCCTT | CTCCCTTTGCAGAACTCAGG |
| Ccl2 | Mus | AGGTCCCTGTCATGCTTCTG | TCTGGACCCATTCCTTCTTG |
| Ccl5 | Mus | ACCACTCCCTGCTGCTTTG | CACTTGCTGCTGGTGTAGAA |
| Acta2 | Mus | CTGACAGAGGCACCACTGAA | CATCTCCAGAGTCCAGCACA |
| Col1a1 | Mus | AAGAGGCGAGAGAGGTTTCC | AGAACCATCAGCACCTTTGG |
| Tgfb1 | Mus | ACCATGCCAACTTCTGTCTGGGAC | ACAACTGCTCCACCTTGGGCTTG |
| Ntn1 | Mus | TTGCAAAGCCTGTGATTGCC | AATCTTGATGCAAGGGGCGA |
| Mmp13 | Mus | TGTTTGCAGAGCACTACTTGAA | CAGTCACCTCTAAGCCAAAGAAA |
| Trem2 | Mus | CAGCACCTCCAGGAATCAAGA | AGGATCTGAAGTTGGTGCCC |
| Timp1 | Mus | CGAGACCACCTTATACCAGCG | ATGACTGGGGTGTAGGCGTA |
| Mmp2 | Mus | CAACGGTCGGGAATACAGCAGC | TGGAAGCGGAACGGGAACTTG |
| Col1a2 | Mus | AGGTCCTAATGGAGATGCCG | CACAGGGCCTTCTTTACCAG |
| Ctgf | Mus | GCTGACCTGGAGGAAAACAT | TGACAGGCTTGGCGATTTTA |
| Col3a1 | Mus | CTGTAACATGGAAACTGGGGAAA | CCATAGCTGAACTGAAAACCACC |
| Adora2b | Mus | ATGCAGCTAGAGACGCAAGAC | GGGATACCAGAAAGTAGTTGGTG |
| Mcam | Mus | ACCTTGAGTTTGAGTGGCTG | CACGTTGTTTAGCTGGAGGA |
| Chd2 | Mus | CGAGTCCAATAGCAGTTCGGA | TGCTTCGATTTGACCTCCTAAC |
| Unc5a | Mus | AGCAGGTCGAGAAAGTGTTTG | GGGCGACAAGGTAGCACAAT |
| Unc5b | Mus | CGGGACGCTACTTGACTCC | GGTGGCTTTTAGGGTCGTTTAG |
| Dcc | Mus | GAAAGACGGCCTTATCCTAGC | GCTTATGGTGTCTGGAATGAAGT |
| Acta2 | Homo | ACTGCCTTGGTGTGTGACAAT G | CGTCACCCACGTAGCTGTCTTT |
| Col1a1 | Homo | AACATGACCAAAAACCAAAAGTG | CATTGTTTCCTGTGTCTTCTG |
| Col1a2 | Homo | GTTGCTGCTTGCAGTAACCTT | AGGGCCAAGTCCAACTCCTT |
| Col3a1 | Homo | GGAGCTGGCTACTTCTCGC | GGGAACATCCTCCTTCAACAG |
| Ctgf | Homo | AGCTGACCTGGAAGAGAACATTAAG | GATAGGCTTGGAGATTTTGGGAGTA |

| ShRNA | Sequence |
| --- | --- |
| pLKO5-GFP-puro-mouse Unc5b-sh1 | AGGTGGAATGGCTCAAGAATG |
| pLKO5-GFP-puro-mouse Unc5b-sh2 | CGCCTACATCGTAAAGAACAA |

| SiRNA | Sequence |
| --- | --- |
| Si-m-NTN1 | GCAGGGCACAAGTCGTATT |
